# *Decreased Vascular Bundle 1* affects mitochondrial and plant development in rice

**DOI:** 10.1186/s12284-021-00454-3

**Published:** 2021-01-25

**Authors:** Lisha Zhang, Ping Feng, Yao Deng, Wuzhong Yin, Yingchun Wan, Ting Lei, Guanghua He, Nan Wang

**Affiliations:** Rice Research Institute, Key Laboratory of Application and Safety Control of Genetically Modified Crops, College of Agronomy and Biotechnology, Academy of Agricultural Sciences, Southwest University, Chongqing, 400715 China

**Keywords:** Plant growth and development, Mic10, Mitochondrial development, Amino acids metabolism, Auxin synthesis, Rice (*Oryza sativa* L.)

## Abstract

**Background:**

Mitochondria are vital regulators of plant growth and development, constitute the predominant source of ATP, and participate in multiple anabolic and catabolic metabolic pathways. But the mechanism by which dysfunctional mitochondria affect plant growth remains unknown, and more mitochondria-defective mutants need to be identified.

**Results:**

A mitochondria-defective mutant *decreased vascular bundle 1* (*dvb1*) was isolated from rice mutant library mutagenized by EMS (ethylmethane sulfonate), which shows dwarfism, narrow leaves, short branches, few vascular bundles, and low fertility. Map-based cloning, genetic complementation, and phylogenetic analysis revealed that *DVB1* encodes a structural protein classified in the Mic10 family and is required for the formation of cristae in mitochondria, and was primarily expressed in vascular bundles. The DVB1 protein is partially localized in the mitochondria and capable of forming dimers and polymers. Comparing with the wild type, disruption of amino acid metabolism and increased auxin synthesis were observed in *dvb1* mutant which also showed increased sensitivity to the mitochondrial electron transport inhibitors.

**Conclusions:**

DVB1 belongs to Mic10 family and DVB1 is partially localized in the mitochondria. Further studies indicated that DVB1 is important for mitochondrial and plant development in rice.

**Supplementary Information:**

The online version contains supplementary material available at 10.1186/s12284-021-00454-3.

## Background

Plant growth and development requires metabolites and energy generated in metabolic processes as regulated by mitochondria. As the predominant site of cellular respiration, mitochondria play a central role in maintaining metabolic and energy homeostasis. In plants, mitochondria constitute an important source of ATP and participate in multiple anabolic and catabolic processes. For example, the tricarboxylic acid (TCA) cycle (the final metabolic pathway in the degradation of sugars, lipids, and amino acids) coupled with oxidative phosphorylation in mitochondria supplies ATPs and carbon skeletons for cells, which are essential for driving plant growth and development. Apart from energy, the roles of mitochondria in a variety of processes, such as amino acid metabolism, hormone biosynthesis, Ca^2+^ homeostasis, regulation of apoptosis, activation of endoplasmic reticulum (ER)-stress response, and intracellular signaling integration are increasingly widely appreciated (Galluzzi et al. 2012; Yee et al. 2014; Berkowitz et al. 2016; Kim et al. 2016; Oxenoid et al. 2016; Van Dingenen et al. 2016). In other words, mitochondria could regulate additional biological processes and promote plant growth and development through the above-mentioned pathways. Thus, mitochondria are vital for growth and development, which is presumably affected when mitochondrial defects occurred. Mitochondrial dysfunction causes a series of typical phenotypes in plants, manifested as sterility, altered stress and cell death tolerance, variegation and albinism, and altered growth and development (Schwarzländer and Finkemeier 2013). However, the exact mechanism of how dysfunctional mitochondria affect plant growth is still unclear.

Mitochondria and auxin are both critical regulators of plant growth and development, and more and more evidence suggest that mitochondrial function and auxin are interconnected. Mitochondrial dysfunction regulates auxin signaling, which in turn which can regulate mitochondrial metabolic and energy pathways to adjust plant growth (Kerchev et al. 2014; Berkowitz et al. 2016). Auxin-associated redox regulation and mitochondria are involved in regulating the establishment and maintenance of the quiescent center of the root apical meristem (Hsieh et al. 2015). In Arabidopsis, mitochondrial retrograde signaling might regulate plant growth and physiological processes through the ER network and auxin signaling (Ivanova et al. 2014). However, the relationship between mitochondria and auxin signaling is still unclear and further study is required.

In plants, auxin is synthesized in cells of vigorously developing tissues, such as the apical meristem, root tips, young leaves and developing seeds, and is involved in apical meristem maintenance, organ primordia formation, and vascular tissue differentiation (Benková et al. 2003; Blilou et al. 2005; Fàbregas et al. 2015). In addition, auxin is important for establishment and maintenance of the vascular cambium, and application of exogenous auxin could induce the formation of additional vascular bundles (Digby and Wareing 1966; Mattsson et al. 1999). In Arabidopsis, the dominant auxin, indole-3-acetic acid (IAA), is synthesized from Trp, Phe, Tyr, Ser, and other aromatic precursors (Benstein et al. 2013; Tivendale et al. 2014). Several tryptophan biosynthesis genes are expressed in vascular tissues, which points to the importance of auxin in vascular bundle development (Birnbaum et al. 2003). Deficiency in auxin synthesis could cause altered tissue development in the panicle, leaf, tiller, coleoptile, and root of rice (Wang et al. 2018).

In our study, we identified a rice mutant *dvb1*, which exhibited dwarfism, narrow leaves, short branches, fewer vascular bundles, and low fertility. *DVB1* encodes a structural protein classified in the Mic10 family, a core subunit of the mitochondrial contact site and cristae organizing system (MICOS) complex, and was partially localized in the mitochondria. In the *dvb1* mutant, the mitochondria exhibited an abnormal structure, amino acid metabolism was disrupted, and the auxin content was increased. The results demonstrated that DVB1 is indispensable in mitochondrial and plant development.

## Results

### Phenotype Characterization of *dvb1* Mutant

**Fig. 1:**
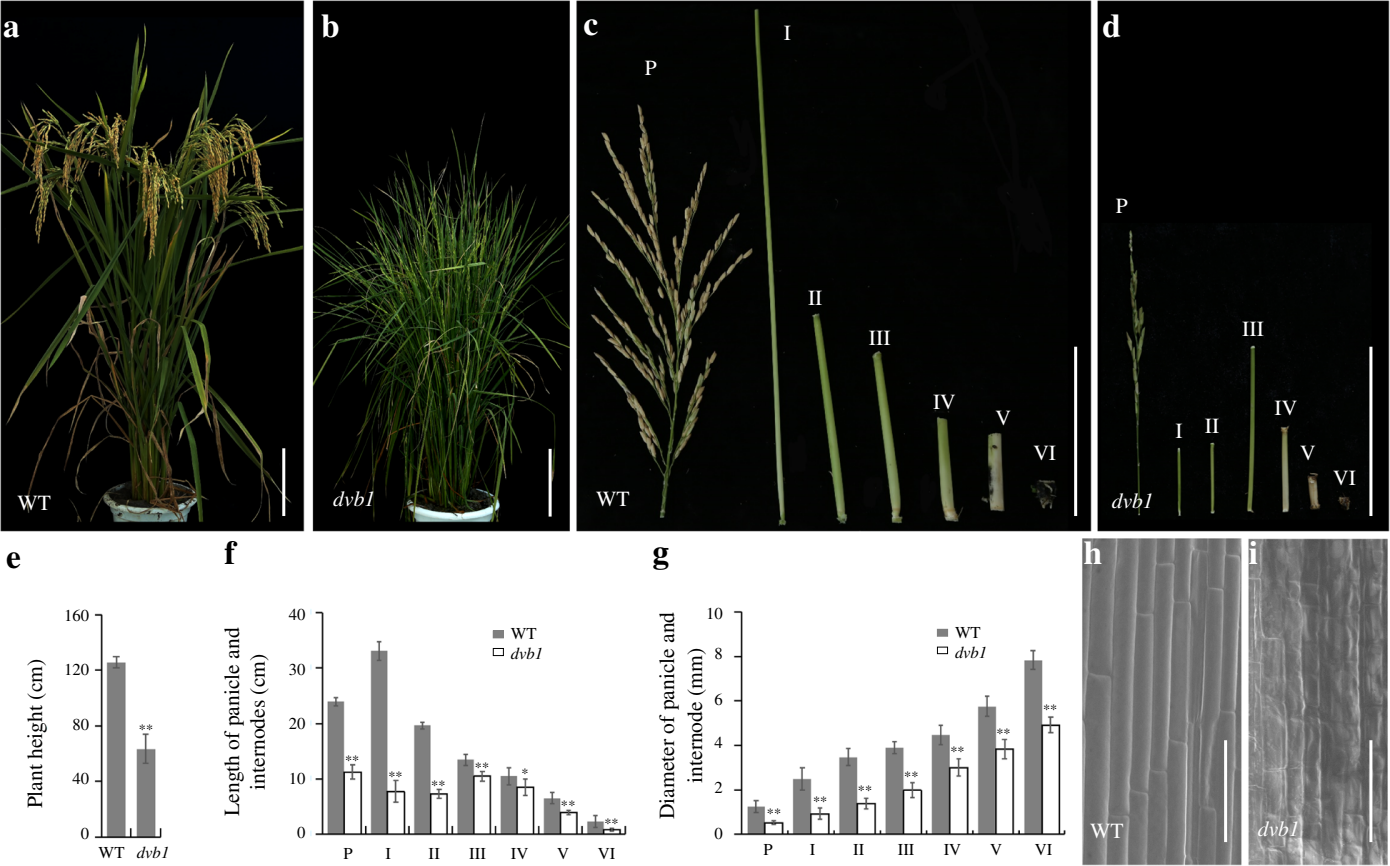
Phenotypes of the *dvb1* mutant and wild type. **a**, **b** Plants of the wild type (**a**) and the *dvb1* mutant (**b**) at maturity. **c**, **d** Panicle and internodes of the wild type (**c**) and *dvb1* (**d**). **e**–**g** Plant height (**e**), panicle and internode lengths (**f**), and diameter (**g**) in the wild type and the *dvb1* mutant. **h**, **i** Scanning electron micrographs of the inner sheath surface in the wild type (**h**) and the *dvb1* mutant (**i**). ‘P’ indicates the panicle and ‘I-VI’ indicate the internodes sequentially from the stem apex to the stem base. Values represent means ± SD from 10 biological samples. Asterisks indicate the significance of differences between the wild type and *dvb1* as determined by Student’s *t*-test (*, 0.01 ≤ *P* < 0.05; **, *P* < 0.01). Scale bars: 15 cm (**a**, **b**), 5 cm (**c**, **d**), 100 μm (**h**, **i**)

Phenotypic differences in the early developmental stages between the wild type and the *dvb1* mutant were relatively indistinct, but the differences became more obvious with the passage of time. At seedling stages, there are no apparent difference in root between the wild-type and the *dvb1* (Additional file 1: Fig.S1 a, c). At booting stage, the *dvb1* root length was significantly shorter than that in the wild-type (Additional file 1: Fig.S1 b, d). At maturity, plants of the *dvb1* mutant were significantly shorter than wild-type plants (Fig. 1a, b). The panicles and internodes of the *dvb1* mutant were significantly thinner and shorter than those of the wild type, especially the first and second ones (Fig. 1c, d). Significant differences in plant height, internode length, and the width of the panicle and internodes were observed between the wild type and the *dvb1* mutant (Fig. 1e–g). Compared with the wild type, the plant height of the *dvb1* mutant was reduced by nearly 50%. The panicle lengths and the first to sixth internodes in the *dvb1* mutant were reduced by 53%, 77%, 65%, 22%, 19%, 40%, and 62%, respectively. The diameters of the panicle base and the first to sixth internodes of the *dvb1* mutant were reduced by 57%, 63%, 60%, 50%, 23%, 34%, and 38%, respectively. Comparing with the wild type, the fertility of the *dvb1* mutant was also affected because of the anthers were abnormal (Additional file 1: Fig. S2 a, b, d, e) with only a small portion of pollen grains stained by KI-I_2_ solution (Additional file 1: Fig. S2 c, f). To clarify whether the cell length or number were changed in the *dvb1* mutant, we examined the sheath inner surface by scanning electron microscopy (SEM) and observed that the cells in the *dvb1* mutant appeared disordered and shorter than those of the wild type (Fig. 1h, i).

One additional obvious difference between the *dvb1* mutant and the wild type was that the leaves of the mutant were narrower (Additional file 1: Fig. S3a), and the degree of narrowing was more significant during later developmental stages (Additional file 1: Fig. S3b). On the 30th, 60th and 90th days after seeds germination, we measured the leaf-width of middle sections of the first, second and third leaves. In contrast to the wild type, the mutated leaf displayed narrower and the degree of narrowing became more distinct with the passage of time (Additional file 1: Fig. S3 c–e). Examination of the wild-type and *dvb1* mutant leaves by SEM and cryosectioning revealed no differences in the leaf surface or vascular bundle spacing (Additional file 1: Fig. S3 f–i).

**Fig. 2:**
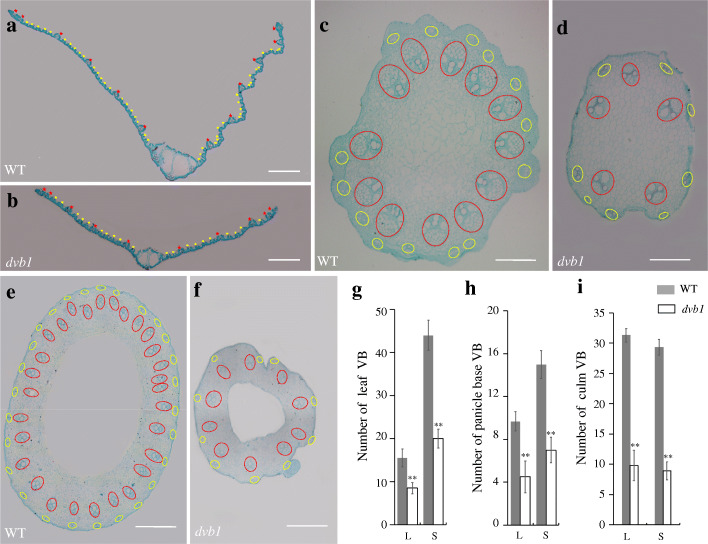
Vascular bundles in the *dvb1* mutant and wild type. **a**–**f** Transverse sections of the leaf (**a**), panicle base (**c**), and culm (**e**) of the wild type, and the leaf (**b**), panicle base (**d**), and culm (**f**) of the *dvb1* mutant. Red labels indicate larger vascular bundles and yellow labels indicate smaller vascular bundles. **g**–**i** Number of vascular bundles in the leaf (**g**), panicle base (**h**), and culm (**i**) of the wild type and the *dvb1* mutant. Values represent means ± SD from 10 biological samples. Asterisks indicate the significance of differences between the wild type and *dvb1* as determined by Student’s *t*-test (*, 0.01 ≤ *P* < 0.05; **, *P* < 0.01). VB, vascular bundles; L, larger vascular bundles; S, smaller vascular bundles. Scale bars: 0.1 mm (**a**–**f**)

Given that the internodes and panicle were thinner, and the leaves were narrower in the *dvb1* mutant, we examined the vascular bundles of the leaf, panicle base, and culm by paraffin sectioning. The number of vascular bundles, both large and small, in these organs was significantly lower in the *dvb1* mutant (Fig. 2a–f). The numbers of large and small vascular bundles were respectively reduced by 55% and 46% in the leaf, 54% and 46% in the panicle base, and 69% and 70% in the culm (Fig. 2g–i). At the booting stage, the numbers of flower primordia and spikelet in the *dvb1* mutant were lower than those in the wild type (Additional file 1: Fig. S4 a–d), which ultimately led to fewer branches in the mature spikes (Additional file 1: Fig. S4 e, f).

### Map-Based Cloning and Functional Complementation of *dvb1*

**Fig. 3:**
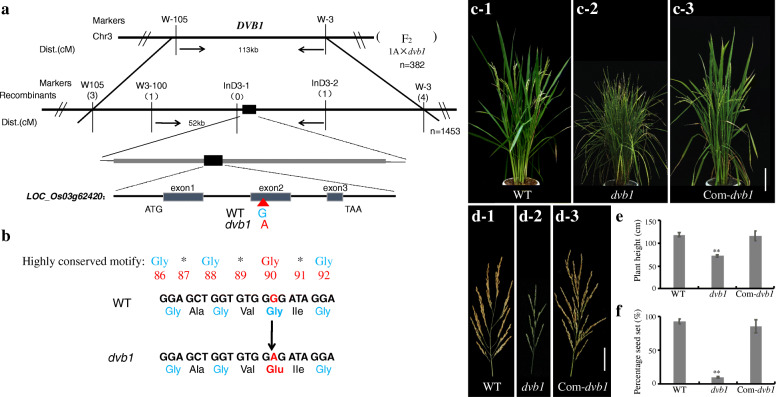
Molecular cloning of *DVB1*. **a** Fine-mapping of *DVB1* on chromosome 3. The thick black line represents the chromosome, the markers used for gene mapping are indicated above the black line, numbers in brackets represent recombinants, the value below the line represents the genetic distance (cM) between two markers, and *n* is the total number of F_2_ genotyped plants. **b** The *dvb1* mutant harbors a nucleotide G-to-A transition in exon 2, leading to a Gly-to-Glu amino acid conversion at site 90 in the core region. **c** Plant of the wild type (c-1), *dvb1* mutant (c-2), *dvb1-*complementary transgenic plants (c-3). Scale bars: 15 cm. **d** Panicle morphology of wild type (D-1), *dvb1* mutant (D-2), *dvb1-*complementary transgenic plants (D-3), Scale bars: 50 mm. **e**–**f** Plant height (**e**) and percentage seed set (**f**) of wild type, *dvb1* mutant, *dvb1-*complementary transgenic plants

To determine whether the phenotypes of the *dvb1* mutant were controlled by a single gene, the *dvb1* mutant was crossed with ‘Xinong 1A’, an indica sterile line bred in Southwest University. All F_1_ individuals exhibited a normal phenotype. The F_2_ generation obtained by self-fertilization of F_1_ individuals showed a segregation ratio of 3:1 (normal: dwarf), which indicates that the mutant phenotypes were controlled by a single recessive nuclear gene. The *DVB1* locus was mapped to a 52 kb region flanked by the markers W3–100 and InD3–2 on chromosome 3. Sequencing analysis revealed a single-nucleotide substitution from G to A in exon 2 within *LOC_Os03g62420* in the *dvb1* mutant, causing an amino acid conversion from Gly-90 to Glu-90 (Fig. 3a, b). Phylogenetic analysis indicated that DVB1 is a member of the Mic10 family, which is conserved in angiosperms, with only one member identified in rice, two members in *Arabidopsis*, and two members in maize, thus the protein function may be highly conserved (Additional file 1: Fig. S5). The Mic10 family of proteins contains a highly conserved motif G*G*G*G, and the mutation site in the *dvb1* mutant was located in the third glycine of this motif (Fig. 3b). To determine whether the phenotypes of the *dvb1* mutant were caused by the mutation of *LOC_Os03g62420*, the *DVB1* complementary vector containing the *DVB1* promoter region and genomic sequence was introduced into the *dvb1* mutant to obtain *dvb1-*complementary transgenic plants. Compared with the wild type and *dvb1* mutant, the positive *dvb1-*complementary transgenic plants showed plant height and panicle development phenotypes similar to those of the wild type (Fig. 3c, d). Similarly, comparison of quantitative morphological traits (plant height and percentage seed set) among the wild type, *dvb1* mutant, and *dvb1-*complementary transgenic plants showed that the wild-type phenotypes were restored in *dvb1-*complementary plants (Fig. 3e, f). These results support the conclusion that *LOC_Os03g62420* was identical to *DVB1*.

### Expression Analysis of *DVB1*

**Fig. 4:**
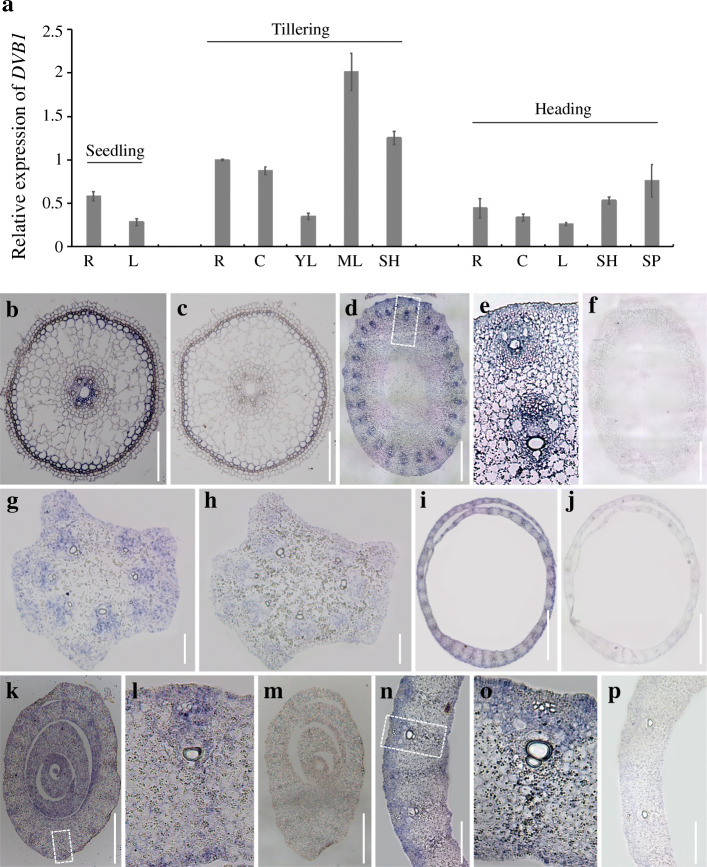
Expression pattern and RNA in situ hybridization of *DVB1.* **a** Abundance of *DVB1* transcripts in different tissues at the seedling, tillering, and heading stages. R, root; L, leaf; C, culm; YL, young leaf; ML, mature leaf; SH, sheath; SP, spikelet. Error bars indicate the standard deviation (SD) and data are means ± SD (*n* = 3). **b**–**p** RNA in situ hybridization of *DVB1* in the root (**b**, **c**), culm (**d**, **f**), panicle base (**g**, **h**), sheath (**i**, **j**), young leaf (**k**–**m**), and mature leaf (**n**–**p**). Images in **e**, **l**, and **o** are local enlargements of **d**, **k**, and **n**, respectively. Images in **b**, **d**, **e**, **g**, **i**, **k**, **l**, **n**, and **o** show the antisense probe of *DVB1*, and images in **c**, **f**, **h**, **j**, **m** and **p** show the sense probe of *DVB1*. Scale bars: 30 μm (**b**, **c**, **d**, **f**, **g**, **h**, **i**, **j**, **k**, **m**, **n**, **p**), 100 μm (**e**, **l**, **o**)

To examine the expression pattern of *DVB1*, we quantified the *DVB1* transcript level in different tissues of the wild type at the seedling, tillering, and heading stages by quantitative real-time PCR (qRT-PCR). *DVB1* transcripts were abundant in the root, culm, leaf, sheath, and spikelet at each stage, and thus showed a non-specific spatiotemporal expression pattern. *DVB1* transcripts showed higher abundance in mature leaves at the tillering stage (Fig. 4a). For more detailed analysis of *DVB1* expression, transverse sections of fixed tissues of the root, culm, leaf, sheath, and panicle base were examined by in situ hybridization. Compared with the negative controls, the strongest signals were observed in the vascular bundles of each tissues (Fig. 4b–p).

### Subcellular Localization of DVB1 Protein

**Fig. 5:**
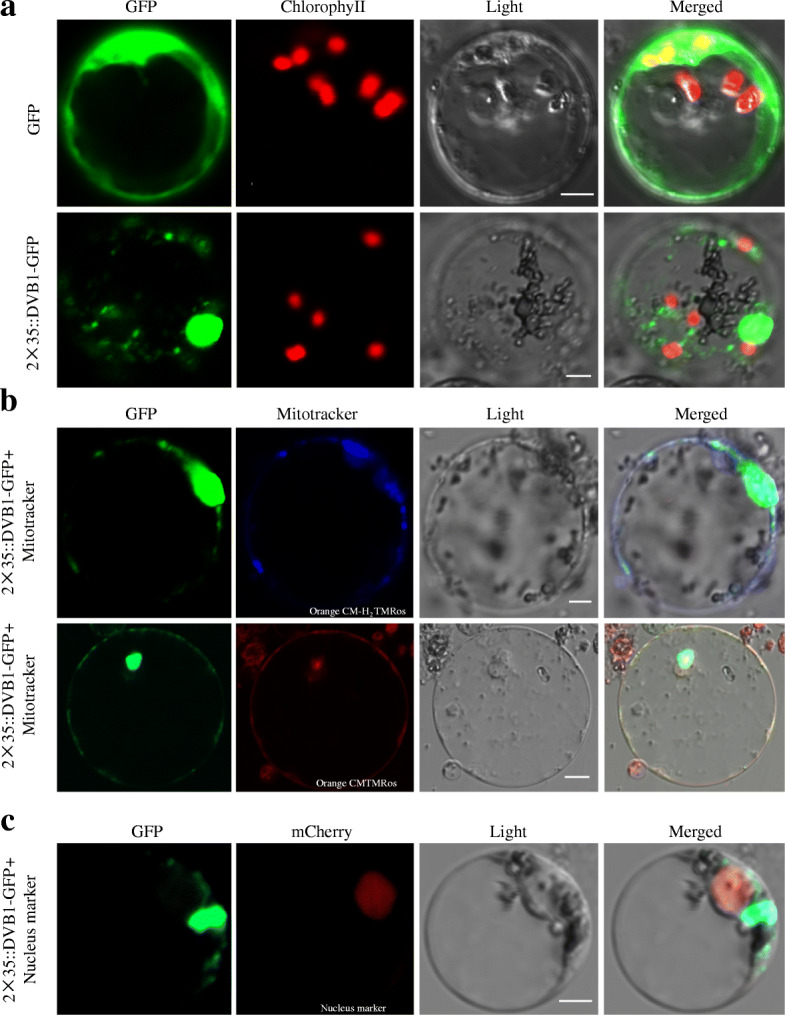
Subcellular localization of DVB1 protein. **a** Subcellular localization of GFP and DVB1-GFP in rice protoplasts. **b** Subcellular localization of DVB1-GFP and MitoTracker (Orange CM-H_2_TMRos and Orange CMTMRos). **c** Subcellular localization of DVB1-GFP and OsH2B-mCherry. Scale bar: 5 μm*.* Scale bar: 5 μm

To examine the subcellular localization of DVB1, the green fluorescence protein (GFP) and the 2 × 35S::DVB1-GFP fusion protein were transiently expressed in rice protoplasts. Green fluorescence from expression of GFP alone was detected uniformly throughout the protoplasts except in the vacuole, whereas the signal from the 2 × 35S::DVB1-GFP fusion protein exhibited a dispersed dot-like and large polymeric cluster-like subcellular distribution (Fig. 5a). We hypothesize that DVB1 was likely to be localized in the mitochondria and nucleus. Protoplasts that expressed the 2 × 35S::DVB1-GFP fusion protein were then stained with Mito-Tracker, and the dot-like signal overlapped with Mito-Tracker staining, which indicates that DVB1 was localized to the mitochondria (Fig. 5b). Next, the DVB1-GFP fusion protein and nuclear marker OsH2B-mCherry were co-expressed in rice protoplasts. The large polymeric cluster-like signal did not overlap with the nuclear marker (Fig. 5c). Subcellular localization of 35S::DVB1-GFP in *Nicotiana benthamiana* leaves revealed a dot-like signal and overlapped with Mito-Tracker staining (Fig. S6). These results suggest that the DVB1 protein was localized in the mitochondria and an undefined cytoplasmic organelle. To detected whether the DVB1^G90E^ localization was changed, 2 × 35S::DVB1^G90E^-GFP was expressed in rice protoplasts. The result showed that DVB1^G90E^ also was located in mitochondria and an undefined cytoplasmic organelle (Fig. S7).

### Mitochondrial Defects in the *dvb1* Mutant

**Fig. 6:**
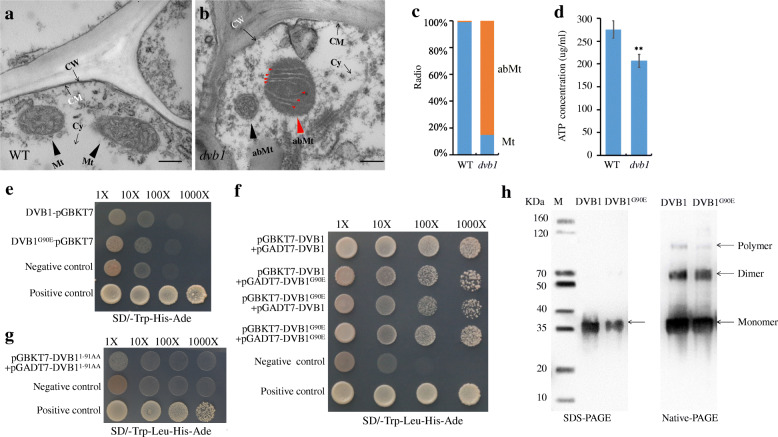
Mitochondrial defect of *dvb1* and dimer and polymer of DVB1 and DVB1^G90E^. **a**, **b** Transmission electron micrographs of mitochondria of the wild type (**a**) and the *dvb1* mutant (**b**). **c** Ratio of normal and abnormal mitochondria in wild type and the *dvb1* mutant. **d** ATP content in wild type and *dvb1* leaves*.* **e** Self-activation of DVB1 and DVB1^G90E^ in yeast cells. (**f**) Interaction with themselves and with each other of DVB1and DVB1^G90E^ in yeast cells. **g**\ Interaction with itself of DVB1^1–91AA^ in yeast cells. **h** Immunoblotting using anti His Tag antibodies after SDS-PAGE (left) showed DVB1 and DVB1^G90E^ expressed and purified from *E. coli* have the expected size of 34.5 kDa, and after Native-PAGE (right) that DVB1 and DVB1^G90E^ can form dimers and smaller degree of polymers. CW: cell wall; CM: cell membrane; Cy: cytoplasm; Mt: normal mitochondria; abMt: abnormal mitochondria; Values represent means ± SD from 6 biological samples. Asterisks indicate the significance of differences between the wild type and the *dvb1* mutant as determined by Student’s *t*-test (*, 0.01 ≤ *P* < 0.05; **, *P* < 0.01). Scale bars: 500 nm (**a**, **b**)

To explore the function of DVB1 in mitochondrial development, we used transmission electron microscopy (TEM) to examine the mitochondrial ultrastructure of the wild type and *dvb1* mutant*.* Compared with the normal mitochondria of the wild type, obvious defects in mitochondrial morphology and cristae organization were observed in the *dvb1* mutant*.* The majority of mitochondria in the *dvb1* mutant were spherical, whereas the wild-type mitochondria were oval or elongated. The observable mitochondria in the *dvb1* mutant exhibited clearly disorganized cristae, which resembled concentric circular structures, whereas the inner membrane of the wild-type mitochondria showed a coiled structure with normal cristae (Fig. 6a, b). The proportions of normal and abnormal mitochondria for the wild type and *dvb1* mutant were scored in 20 sections (Fig. 6c). Mitochondria are sites of oxidative metabolism in eukaryotes, where energy from ATP molecules is released. We therefore measured the ATP content in the *dvb1* mutant and the wild type as important indicators of mitochondrial functioning. Compared with the wild type, the ATP content was significantly lower in the *dvb1* mutant (Fig. 6d, e), which implies that the ultrastructural abnormalities in the *dvb1* mutant affected mitochondrial functions.

The yeast Mic10, as the second MICOS core component, is a small hairpin topology membrane protein with two transmembrane domains consisting of hydrophobic amino acids. The Mic10 subunit could form oligomers and be shaped into a hairpin topology through a large number of conserved glycine sequences (G*G*G*G), and could bend the inner membrane of mitochondria to form cristae (Bohnert et al. 2015; Rampelt et al. 2017). We thus tested whether OsDVB1 can form oligomers as in yeast, by using yeast two-hybrid assay, DVB1 and DVB1^G90E^ could interact with themselves and with each other (Fig. 6f, g). In addition, the anterior protein (DVB1^1–91AA^), which did not contain the mutation site of the *DVB1* gene, was unable to interact with itself in a yeast two-hybrid assay (Fig. 6h). We produced His-tagged DVB1 and DVB1^G90E^ in *Escherichia coli* and purified the proteins. The results of Western blotting and Native-PAGE confirmed that DVB1 and DVB1^G90E^ can form a dimer and, to a lesser degree, polymers (Fig. 6i). Thus, DVB1 presumably regulate mitochondrial development in rice by the same mechanism as Mic10 in yeast. However, in contrast to yeast, DVB1^G90E^, which contained the mutation in the conserved motif G*G*G*G, could also form dimers and a small proportion of polymers.

### *DVB1* Influences Amino Acid Metabolism and Auxin Synthesis

**Fig. 7:**
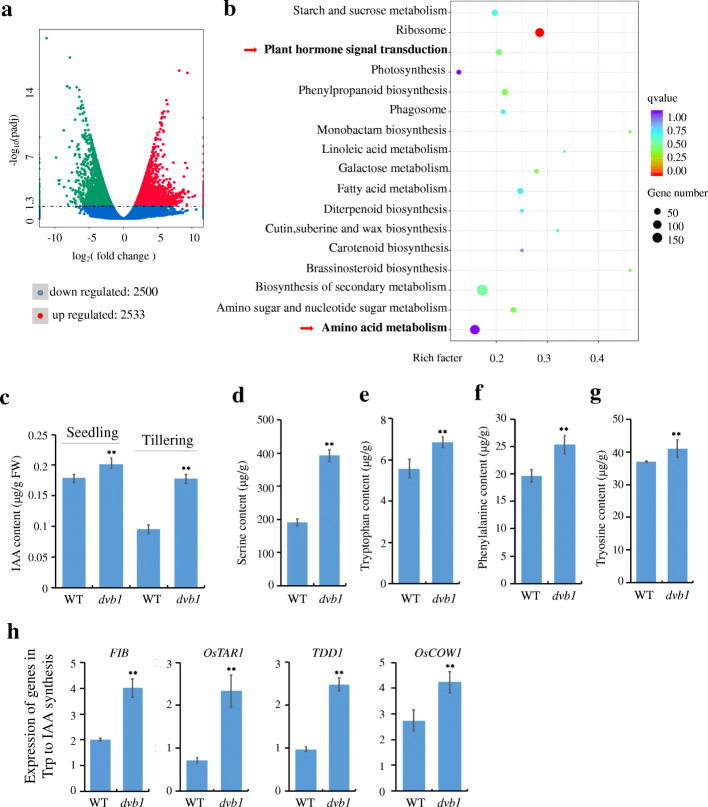
*DVB1* involvement in the auxin synthesis pathway. **a**, **b** Volcano plot of genes differentially expressed in leaves at tillering stage between the *dvb1* mutant and the wild type (**a**) and KEGG pathway enrichment scatterplot of differentially expressed genes (**b**) from RNA-sequencing data. **c** IAA content of leaves at seedling and tillering in the wild type and *dvb1* plants*.* **d**-**g** Serine (**d**), tryptophan (**e**), phenylalanine (**f**) and tyrosine (**g**) contents of leaves at tillering stage in the wild type and *dvb1* plants. **h** Expression of genes involved in auxin synthesis of leaves at tillering stage in the wild type and *dvb1.* Values represent means ± SD from 6 biological samples. Asterisks indicate the significance of differences between the wild type and *dvb1* as determined by Student’s *t*-test (*, 0.01 ≤ *P* < 0.05; **, *P* < 0.01)

To explore further function of DVB1, we performed RNA sequencing (RNA-Seq) assays to evaluate differential gene expression between the *dvb1* mutant and the wild type. The RNA-Seq data from leaves at the tillering stage revealed 2533 up-regulated and 2500 down-regulated genes in the *dvb1* mutant compared with the wild type, using the threshold of twofold change and a Student’s *t*-test significance cutoff of *P* < 0.05 (Fig. 7a). Kyoto Encyclopedia of Genes and Genomes (KEGG) pathway enrichment analysis revealed that the genes differentially expressed between the *dvb1* mutant and the wild type were predominantly enriched in certain pathways, which included plant hormone signal transduction and amino acid metabolism (Fig. 7b).

The *dvb1* mutant phenotypes, namely dwarfism, narrow leaves, short branches, fewer vascular bundles, and abortive pollen, all these suggests that the *dvb1* mutant was affected by auxin deficiency (Wang et al. 2018). Taken that genes differentially expressed between the *dvb1* mutant and the wild type involved in plant hormone, and mitochondrial morphology and function were defective in the *dvb1* mutant, this led us to examine whether the auxin content was changed in the *dvb1* mutant. Therefore, we measured the IAA content of leaves at the seedling and tillering stages. The IAA content in the *dvb1* mutant was significantly increased by 13.02% at the seedling stage and 86.3% at the tillering stage compared to the wild type (Fig. 7c).

Mitochondria are involved in amino acid metabolism (Mcbride et al. 2006; Berkowitz et al. 2016). Reduced influx of amino acids in the TCA cycle leads to an increase in amino acid contents and decrease in ATP production, causing severe growth penalties in the root and shoot (Yu et al. 2012). Some specific amino acids are precursors for conversion of Trp to IAA. Subsequently, we measured the amino acid (Ser, Trp, Phe, and Tyr) contents of several auxin precursors. Ser, Trp, Phe, and Tyr contents were increased by 105.3%, 22.8%, 28.6%, and 10.63%, respectively, in the *dvb1* mutant compared with those of the wild type (Fig. 7d–g). Four genes, namely *OsFIB*, *OsTAR1*, *OsTDD1*, and *OsCOW1*, play a crucial role in the conversion of Trp to IAA in rice (Woo et al. 2007; Sazuka et al. 2009; Yoshikawa et al. 2014). Thus, we detected their relative expression level in the *dvb1* mutant and the wild type by qRT-PCR. Those four genes showed higher relative expression levels in the *dvb1* mutant compared with those of the wild type (Fig. 7h). These indicate that Trp-to-IAA synthesis in the *dvb1* mutant was defective.

### Mitochondria Affect Auxin Synthesis

**Fig. 8:**
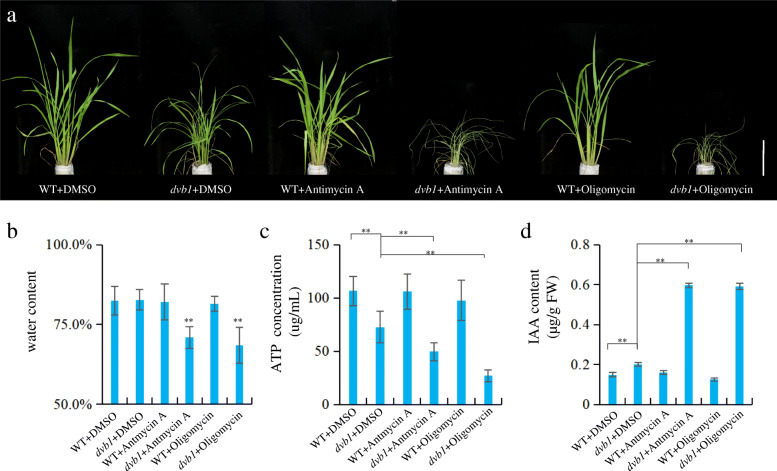
Treatment with mitochondrial electron transport inhibitors. **a** Phenotypes of the wild type and the dvb1 mutant after treatment with 1.25 μM antimycin A and 0.5 μM oligomycin. The DMSO was as a control. Scale bars: 15 cm. **b**–**d** The content of water (**b**), ATP (**c**), IAA (**d**) of the wild type and the dvb1 mutant after treatment with 1.25 μM antimycin A and 0.5 μM oligomycin. Values represent means ± SD from 6 biological samples. Asterisks indicate the significance of differences between the wild type and dvb1 as determined by Student’s t-test (*, 0.01 ≤ *P* < 0.05; **, *P* < 0.01)

To further explore the relationship between mitochondria and auxin synthesis, we treated the wild type and *dvb1* mutant with antimycin A and oligomycin. Antimycin A and oligomycin are inhibitors of cytochrome *c* reductase, thereby inhibiting electron transport from ubiquinone to cytochrome *c*, which may decrease ATP contents in mitochondria and chloroplasts (Stoimenova et al. 2007; Nishikawa et al. 2012). Compared with untreated plants, no obvious differences were observed in treated wild-type plants, whereas the *dvb1* mutant treated with antimycin A and oligomycin showed severe growth defects, withering and died after a few days’ treatment, which indicates that the *dvb1* mutant was more sensitive to antimycin A and oligomycin (Fig. 8a). In treated wild-type plants, the water, ATP and IAA content seemed to remain unchanged comparing with the untreated ones. While in treated *dvb1* plants, the water and ATP content decreased, and the IAA content increased significantly compared with untreated *dvb1* plants (Fig. 8b–d). These results imply that the mitochondria of the *dvb1* mutant was indeed defective which leads to IAA content was significantly increased.

## Discussion

The mitochondrion is a double-membrane-bound organelle comprising a relatively smooth outer membrane and a highly folded inner membrane. The inner membrane can be further divided into two parts: the inner boundary membrane parallel to the outer membrane, and the mitochondrial cristae formed by invaginations of the inner membrane to increase the membrane surface area for adapting to mitochondrial function (Wurm and Jakobs 2006). Mitochondrial structure and functions are highly conserved among organisms and during cristae formation, a member of the MICOS complex typically folds the inner membrane to the mitochondrial matrix. The Mic10 subunit in yeast, a core member of the MICOS complex, is a small hairpin-like membrane protein containing two transmembrane domains. In addition, the Mic10 protein contain a conserved G*G*G*G motif and can be shaped into oligomers to induce membrane curvature into cristae junctions through the G*G*G*G sequence (Russ and Engelman 2000; Alkhaja et al. 2012). DVB1 is a member of the Mic10 family, of which only one member has been identified in rice. Subcellular localization in rice protoplasts and *N. benthamiana* leaves showed that DVB1 was localized in mitochondria. The mutation of DVB1^G90E^ was located in the G*G*G*G sequence, but DVB1^G90E^ was still capable to forming a dimer and polymer and the localization of DVB1^G90E^ was not changed with DVB1. These results implied that the rice Mic10 protein functions in the form of a polymer, as in yeast, but may have undergone changes.

Respiratory chain complexes and F_1_F_0_-ATP synthase adhere to the mitochondrial cristae and work together to produce ATP and electron delivery (Gilkerson et al. 2003). The mitochondria in the *dvb1* mutant were abnormally developed, and the ATP content was significantly lower than those in the wild type. Therefore, DVB1 is important for determination of mitochondrial morphology and function. Amino acids are important substrates for TCA cycle intermediates and mitochondrial morphological and structural defects may directly regulate the TCA cycle, which can affect the metabolism of amino acids (Cogliati et al. 2013; van der Bliek et al. 2017; Bröer and Bröer 2017). Functionally defective mitochondria were observed in the *dvb1* mutant, and the contents of Ser, Trp, Phe, and Tyr were significantly increased in the *dvb1* mutant as a result. These amino acids enter the Trp-dependent IAA synthesis pathway in the form of Trp, which is a critical pathway for IAA biosynthesis (Sazuka et al. 2009). By detecting the expression of four enzymes involved in the conversion of Trp to IAA, namely *OsFIB*, *OsTAR1*, *OsTDD1*, and *OsCOW1*, we showed that the Trp-dependent IAA biosynthesis pathway was strengthened in the *dvb1* mutant. Ultimately, the IAA content in the leaves of the *dvb1* mutant was increased compared with the wild type, and this increase was greater at the tillering stage than that at the seedling stage, suggesting that auxin synthesis was enhanced in the *dvb1* mutant. Both mitochondrial and auxin synthesis defects may cause abnormal plant growth and development. The *dvb1* mutant is a dwarfed plant, with shorter internodes, narrower leaves, fewer flower primordia and branches, fewer vascular bundles, and lower fertility. Compared with the phenotypic changes caused by mitochondrial defects, the *dvb1* mutant phenotype showed similarities to a mutant associated with auxin deficiency (Wang et al. 2018). Treatment of wild-type and *dvb1* mutant plants with antimycin A and oligomycin resulted in a withered appearance in the *dvb1* mutant but not the wild type at the same developmental stage. The ATP content changed to a greater extent in the *dvb1* mutant after treatment, which further implies that the mitochondria of the *dvb1* mutant were defective and more sensitive to antimycin A and oligomycin. In addition, the IAA content of the treated *dvb1* mutant plants was significantly increased compared with untreated *dvb1* mutant plants, which suggests to some extent that mitochondrial abnormalities may affect IAA metabolism.

Mitochondria are present in almost all eukaryotic cells and are involved in multiple basic metabolic processes. Increasing evidence points to a relationship between mitochondria and auxin. The Arabidopsis *slo3* mutant, which shows retarded root growth and curly rosette leaves, together with other mitochondrial mutants showing reduced growth rates and a short root phenotype, provide insight into the interactions between dysfunctional mitochondria and auxin signaling (Hsieh et al. 2015). In the present study, we show that *DVB1* encodes a Mic10 family protein that affects mitochondrial development and IAA synthesis, which to some extent provides evidence for the mitochondrial impact on IAA and brings novel insights into the function of Mic10 in plants. More importantly, the *dvb1* mutant, with its dwarfed habit, shorter internodes, narrower leaves, fewer vascular bundles, and reduced fertility exhibits a phenotype consistent with typical auxin-related and mitochondrial-related deficiency, and both mitochondria and IAA are defective in the *dvb1* mutant. Thus, the *dvb1* mutant may play an important role in elucidating the connection between these two deficiencies.

Mic60 is another core subunit of the MICOS complex and also plays an important role in cristae formation. The AtMic60 protein participates in a large mitochondrial transmembrane lipoprotein complex responsible for the transport of mitochondrial lipids (Michaud et al. 2016). In the present study, in addition to cristae formation, DVB1 influenced amino acid metabolism and IAA synthesis, combining the role of AtMic60 in transporting lipids, which suggests that different Mic proteins in the MICOS complex might perform additional physiological functions in cells except the maintenance of mitochondria. Nevertheless, it is clear that DVB1 performs important and specializes functions in mitochondrial and plant development.

## Conclusion

A mitochondria-defective mutant (*dvb1*) was identified in rice, showing dwarfism, narrow leaves, short branches, few vascular bundles, and low fertility. *DVB1* encodes a structural protein classified in the Mic10 family and is required for the formation of cristae in mitochondria, and is primarily expressed in vascular bundles. The DVB1 protein is partially localized in the mitochondria. Both DVB1 and DVB1^G90E^ are capable to forming dimers and polymers. Disruption of amino acid metabolism and increased auxin synthesis were observed in the *dvb1* mutant. Compared with the wild type, the *dvb1* mutant showed increased sensitivity to the mitochondrial electron transport inhibitors. Results from this work suggest that *DVB1* plays a crucial role in mitochondrial and plant development in rice.

## Materials and Methods

### Plant Materials

We treaded the Jinhui 10 seeds (JH10) (*Oryza sativa* L. *ssp. indica*) with 1% ethylmethane sulfonate. In T_1_ generation, the *dvb1* was found and was inherited stably after more than three years of observation. JH10, a restorer, was used as the wild-type (WT) in all experiments, all plants were bred in Southwest University, Chongqing, China, under natural conditions.

### Characterization of Mutant Phenotypes

The phenotypes of the *dvb1* mutant and the wild type were compared over the entire growth period. Agronomic traits, including root length, plant height, and length and width of the panicle and internodes, were measured. The wild type and *dvb1* mutant plants were photographed using a Canon 5DIII digital camera (Tokyo, Japan). The inner surface of the sheath was observed using a SU3500 scanning electron microscope (Hitachi, Japan) at an accelerating voltage of 5.0 kV at − 4 °C.

### Paraffin Sectioning, Cryosectioning, and Histological Analysis

Leaves, panicle base, and culms were collected at the tillering stage from wild type and *dvb1* mutant plants, and fixed in 50% ethanol, 0.9 M acetic acid, and 3.7% formaldehyde overnight at 4 °C. The fixed samples were dehydrated with a graded series of ethanol (50%, 70%, 85%, 95%, 100%, 100%, and 100%), infiltrated with xylene, and embedded in paraffin (Sigma, USA). A rotary microtome RM2245 (Leica, Germany) was used to cut 8-μm-thick sections, which were transferred onto poly-L-lysine coated glass slides, deparaffinized in xylene, dehydrated through an ethanol series, and stained with safranin-O and fast green. For cryosectioning, wild-type and *dvb1* mutant leaves were cut into 0.5 cm pieces, placed in an embedding agent ENG50 (Thermo, USA), and rapidly frozen. The frozen samples were sliced to about 10 μm thickness, stained in hematoxylin and eosin solution, then dehydrated through a gradient ethanol series (30%, 50%, 70%, 85%, 95% and 100%). The sections were observed using an Eclipse E600 light microscope (Nikon, Japan).

### Genetic Analysis and Fine-Mapping of *DVB1*

The *dvb1* mutant was crossed with rice ‘Xinong 1A’ (bred by the Southwest University Rice Research Institute) to generate the F_1_ population; the F_2_ population was derived by self-fertilization of the F_1_ population. The F_2_ individuals that exhibited the mutant phenotype were selected and used to map *DVB1*. Gene fine-mapping was conducted using simple sequence repeat markers obtained from the publicly available rice databases Gramene (http://www.gramene.org) and the Rice Genomic Research Program (http://rgp.dna.affrc.go.jp/E/publicdata/caps/index.html). Insertion/deletion markers were developed from comparison of the genomic sequences of ‘Xinong 1A’ and ‘Jinhui 10’.

### Phylogenetic Tree Construction

Sequences of DVB1 homologous proteins from other plant species were acquired by conducting a BLAST search of the Phytozome portal (v12.1.6) (http://Phytozome.jgi.doe.gov/pz/portal.html#!Search?show=BLAST). Vector NTI Advance 10 was used to align the amino acid sequences. The alignment was saved as a FASTA file and used for phylogenetic analysis with MEGA (v5.2). A phylogenetic tree was constructed using the neighbour-joining method based on the evolutionary model with the lowest Bayesian information criterion score. Bootstrap support values from 1000 replicates are presented for each node. The peptide sequences used for phylogenetic tree construction are listed in Supplementary Table S1.

### Complementary Vector Construction of *DVB1*

To complement the mutant phenotype of *dvb1*, the 4215 bp genomic fragment that contained the *DVB1* coding sequence, coupled with 1874 bp upstream (including the transcription promoter) and 450 bp downstream sequences, was amplified from the wild-type genomic DNA and cloned into the binary vector pCAMBIA1301. The recombinant complementation plasmids were introduced into the *dvb1* mutant using the *Agrobacterium tumefaciens*-mediated method. The primer sequences used for vector construction are listed in Supplementary Table S2.

### RNA Extraction and Quantitative Real Time PCR Analysis

Total rice RNA was extracted from dissected tissues of the root, culm, leaf sheath, leaf, and panicle, and purified using the RNAprep Pure Plant Kit (Tiangen, China) in accordance with the manufacturer’s instructions. The concentration, purity, and integrity of extracted RNA were determined using a NanoDrop 2000 spectrophotometer (Bio-Rad, China) and 1% agarose gel electrophoresis. All reverse transcriptions were performed using 1 μg total RNA with SuperScript® III Reverse Transcriptase (Invitrogen, China) employing the oligo(dT) 18 primer in accordance with the manufacturer’s instructions. After cDNA synthesis, all samples were diluted tenfold with sterilized water, and qRT-PCR analysis was performed with the SYBR Supermix Kit (Bio-Rad, China) and the ABI 7500 Sequence Detection System (Thermo Fisher, USA) in accordance with the manufacturer’s instructions. Three replicates were performed and normalized relative expression levels were calculated by the ∆∆*C*_*t*_ method using *ACTIN1* as an endogenous control (Livak and Schmittgen 2001). The primers used are listed in Supplementary Table S2.

### In Situ Hybridization

The 204 bp specific probe for *DVB1* was amplified and labelled using the DIG RNA Labelling Kit (Roche, Switzerland) in accordance with the manufacturer’s recommendations. Pretreatment of sections, hybridization, and immunological detection were performed as described previously (Ma et al. 2017). The primer sequences used are listed in Supplementary Table S2.

### Subcellular Localization in Rice Protoplasts

The full-length coding region of *DVB1* without the stop codon was amplified and cloned into the Pan580 vector driven by double 35S promoter (2 × 35S promoter) to generate the 2 × 35S::DVB1-GFP (green fluorescent protein) fusion protein. Both Pan580-DVB1-GFP and empty Pan580-GFP plasmids were introduced into rice protoplasts as described previously (Ma et al. 2017). After incubation at 28 °C overnight, GFP fluorescence was observed using a LSM 780 confocal laser scanning microscope (Zeiss, Germany). Protoplasts were incubated with 0.5 mM MitoTracker™ Orange CM-H_2_TMRos and MitoTracker™ Orange CMTMRos (Invitrogen, China) for 30 min to stain the mitochondria (Wang et al. 2016). To explore whether subcellular distribution of DVB1 overlapped with a nuclear marker, DVB1-GFP and OsH2B-mCherry plasmids were co-introduced into rice protoplasts (Guo et al. 2018). To check DVB1^G90E^’s location, the full-length coding region of DVB1^G90E^ without the stop codon was amplified and cloned into the Pan580 vector driven by double 35S promoter (2 × 35S promoter) to generate the 2 × 35S:: DVB1^G90E^-GFP (green fluorescent protein) fusion protein. The transformation method is as above. The primer sequences used are listed in Supplementary Table S2.

### Subcellular Localization in *Nicotiana benthamiana*

The full-length coding sequence of DVB1 was cloned into the pCAMBIA1300 vector driven by single 35S promoter to generate the 35S::DVB1-GFP fusion protein. Plasmids were introduced into leaf cells of *Nicotiana benthamiana* using an *Agrobacterium* (strain GV3101)-mediated infiltration. The pCAMBIA1300 vector was infiltrated as a control. After infiltration for 36–56 h, leaf cells were incubated with 0.5 mM MitoTracker™ Orange CMTMRos (Invitrogen, China) for 30 min to stain the mitochondria (Tabatabaei et al. 2019). The GFP and MitoTracker signals were visualized using a LSM 780 confocal laser scanning microscope (Zeiss, Germany). The primer sequences used are listed in Supplementary Table S2.

### Transmission Electron Microscopy

Tissue fixation and preparation for TEM followed a previously described method with minor modifications (Scafaro et al. 2011). Leaves at the tillering stage were collected and cut into 1 mm^2^ squares, fixed in fixative solution (3.5% glutaraldehyde, 2% paraformaldehyde, and 0.1 M phosphate buffer) for at least 48 h, and then post-fixed in 1% osmium tetroxide for 2 h after washing with 0.1 mol L^− 1^ phosphate saline buffer. Tissues were stained with uranyl acetate, dehydrated in ethanol, and embedded in Spurr’s medium before thin sectioning. Samples were stained again and mitochondria examined using a H-7500 transmission electron microscope (Hitachi, Japan).

### Measurement of ATP Content

ATP extraction was performed as previously described (Zeng et al. 2013). Leaf tissue (0.1 g fresh weight [FW]) sampled at the tillering stage was homogenized in liquid nitrogen, mixed immediately with 400 μL of 5 M perchloric acid, and boiled for 10 min in a water bath. The solution was centrifuged at 16,000 *g* for 15 min at 4 °C. The clear supernatant was collected for ATP estimation by high-performance liquid chromatography (HPLC) using an ACQUITY™ UPLC™ BEH-C18 column (2.1*100 mm, 1.7 μm; Waters, USA) and H-Class UPLC™ Tunable UV Detector (Waters, USA).

### Yeast Two-Hybrid Assay

The Matchmaker™ GAL4 Two-Hybrid System (Clontech, China) was used to determine whether the DVB1 and DVB1^G90E^ proteins could form homotypic oligomers. The full-length DVB1 and DVB1^G90E^ coding sequences were cloned into the pGAD-T7 and pGBK-T7 vectors, respectively. The 1–237 bp sequence of *DVB1* (lacking the *DVB1* mutation site) was cloned into the pGAD-T7 and pGBK-T7 vectors, and transformed into yeast cells. The DO-Leu-His-Ade and DO-Leu-Trp-His-Ade filter assays were performed in accordance with the manufacturer’s instructions to check self-activation of DVB1 BD and DVB1^G90E^ BD and protein–protein interactions.

### Gel Electrophoresis and Immunoblot Analysis

To produce a His-tagged protein, the full-length DVB1 and DVB1^G90E^ coding sequences were cloned into the expression vector pET-32a (Novagen, China) using the *Kpn*1 and *Eco*R1 restriction enzymes, and introduced into *E. coli* strain BL 21 (DE3). Positive colonies were grown at 37 °C to an optical density at 600 nm (OD_600_). The fusion protein was induced by addition of isopropyl.

β-D-thiogalactopyranoside to a final concentration of 1 mM, and then was purified with Ni-NTA Sefinose™ Resin (BBI, China) following the manufacturer’s protocol. Immunoblot analysis by SDS-PAGE and Native PAGE using a His-tag antibody (Proteintech, USA) were performed as described previously (Fujita et al. 2003).

### Transcriptome Analysis

Leaves of wild-type and *dvb1* mutant plants were sampled at the tillering stage for transcriptome sequencing experiments with three biological replicates (Allwegene Company, China). Total RNA was extracted from the samples using the TRIzol Reagent (Invitrogen, China) in accordance with the manufacturer’s instructions. RNA sequencing was conducted on an Illumina HiSeq platform. The RNA-Seq clean reads were aligned to the TIGA rice genome using TopHat (v2.1.0). The RNA-Seq data were analysed using a previously described method. Differentially expressed genes were detected using the DESeq R package (v1.10.1) with a relative change threshold of two (*P* < 0.05, false discovery rate < 0.01). Gene ontology (GO) categories were identified using the GOseq R package. KEGG pathways were assigned using the KEGG database (http://www.kegg.jp/) and were considered significant at *P* < 0.05.

### Free IAA Measurement

Leaf tissue (100 fresh weight [FW]) sampled at the tillering stage was homogenized in liquid nitrogen, then 1 mL precooled sodium phosphate buffer was added and the homogenate was incubated at 4 °C overnight in the dark. After centrifugation at 8000 *g* for 10 min, the residue was diluted with 0.5 mL precooled sodium phosphate buffer for 2 h and then evaporated under N_2_ gas at 40 °C until the organic phase had disappeared. Petroleum ether (0.5 mL) was added to extract the residue three times, and the lower layer was evaporated to dryness under N_2_ gas at 40 °C. The residue was dissolved in 0.5 mL of the mobile phase (600 mL ultrapure water, 6 mL acetic acid, and 400 mL methanol) and then filtered. Free IAA in the extract was analysed using an Agilent 1100 HPLC (Agilent, USA) and Kromasil C18 reversed-phase column (250 mm * 4.6 mm, 5 μm; Waters, USA) (Krisantini et al. 2009).

### Extraction and Measurement of Amino Acids

Leaf tissue (10 mg; FW) sampled at the tillering stage was homogenized in liquid nitrogen and extracted with 1 mL precooled extraction mixture (1% formic acid–methanol) for 10 min at 25 °C. After centrifugation at 13,000 *g* at 4 °C for 5 min, the supernatant was diluted fivefold. An aliquot (100 μL) of diluted supernatant was added to 100 μl of 100 ppb double isotope internal standard (Phe-1-^13^C and Val-1-^13^C) and vortexed for 30 s. After filtration, the supernatant was analysed by liquid chromatography–mass spectrometry (LC/MS) with regard to amino acid standards. An ACQUITY™ UPLC™ BEH C18 column (2.1 × 100 mm, 1.7 μm; Waters, USA) and electrospray positive ionization (ESI, USA) source was used for LC/MS (Liyanaarachchi et al. 2017).

### Treatment with Mitochondrial Electron Transport Inhibitors

The antimycin A (ShangHai Maokang Biotechnology, China) and oligomycin (ShangHai Maokang Biotechnology, China) used were from acqueous dilutions of DMSO stock solutions (150 mM antimycin A; 20 mM oligomycin) to 1.25 μM antimycin A and 0.5 μM oligomycin. Wild-type and *dvb1* mutant plants were treated with the inhibitor for about 4 days. The corresponding solution of water mixed with DMSO was used as the control. After treatment, the water, ATP and IAA contents were measured and analysed. The treated plants were photographed using a Canon 5DIII digital camera (Tokyo, Japan). All experiments were performed using at least six independent biological replicates.

### Accession Numbers

Sequence data from this article can be found in the GenBank/EMBL libraries under the following accession numbers: *OsDVB1* (LOC_03g62420), *OsFIB* (LOC_01g07500), *OsTAR1* (LO05g07720), *OsTDD1* (LOC_04g38950), *OsCOW1* (LOC_03g06654).

## Supplementary Information

**Additional file 1: Fig. S1.** Root length of WT and *dvb1*. **Fig. S2.** Anthers and pollens in *dvb1*. **Fig. S3.** Narrowed leaves in *dvb1*. **Fig. S4.** Flower primordia and branches in *dvb1*. **Fig. S5.** Phylogenetic tree of DVB1. **Fig. S6.** Subcellular localization of DVB1 protein in *Nicotiana benthamiana*. **Fig. S7.** Subcellular localization of DVB1^G90E^. **Table S1.** Peptide sequences used for constructing the NJ tree. **Table S2.** Primer sequences used in this study.

## Data Availability

The data sets supporting the conclusions of this article are included within the article and its additional files.

## Abbreviations

EMS: Ethylmethane sulfonate
TCA: Tricarboxylic acid
ER: Endoplasmic reticulum
IAA: Indole-3-acetic acid
MICOS: Mitochondrial contact site and cristae organizing system
SEM: Scanning electron microscopy
GFP: Green fluorescence protein
qRT-PCR: Quantitative real-time PCR
TEM: Transmission electron microscopy
