# Supplementary material for: Decreased Vascular Bundle 1 affects mitochondrial and plant development in rice
Source: Rice (N Y). 2021 Jan 25;14:13. doi: 10.1186/s12284-021-00454-3 (PMC7835275; doi:10.1186/s12284-021-00454-3)
Supplement: Supplementary file 1 — Additional file 1: Fig. S1. Root length of WT and dvb1. Fig. S2. Anthers and pollens in dvb1. Fig. S3. Narrowed leaves in dvb1. Fig. S4. Flower primordia and branches in dvb1. Fig. S5. Phylogenetic tree of DVB1. Fig. S6. Subcellular localization of DVB1 protein in Nicotiana benthamiana. Fig. S7. Subcellular localization of DVB1G90E. Table S1. Peptide sequences used for constructing the NJ tree. Table S2. Primer sequences used in this study. [file 12284_2021_454_MOESM1_ESM.doc]

**Supplementary Data**


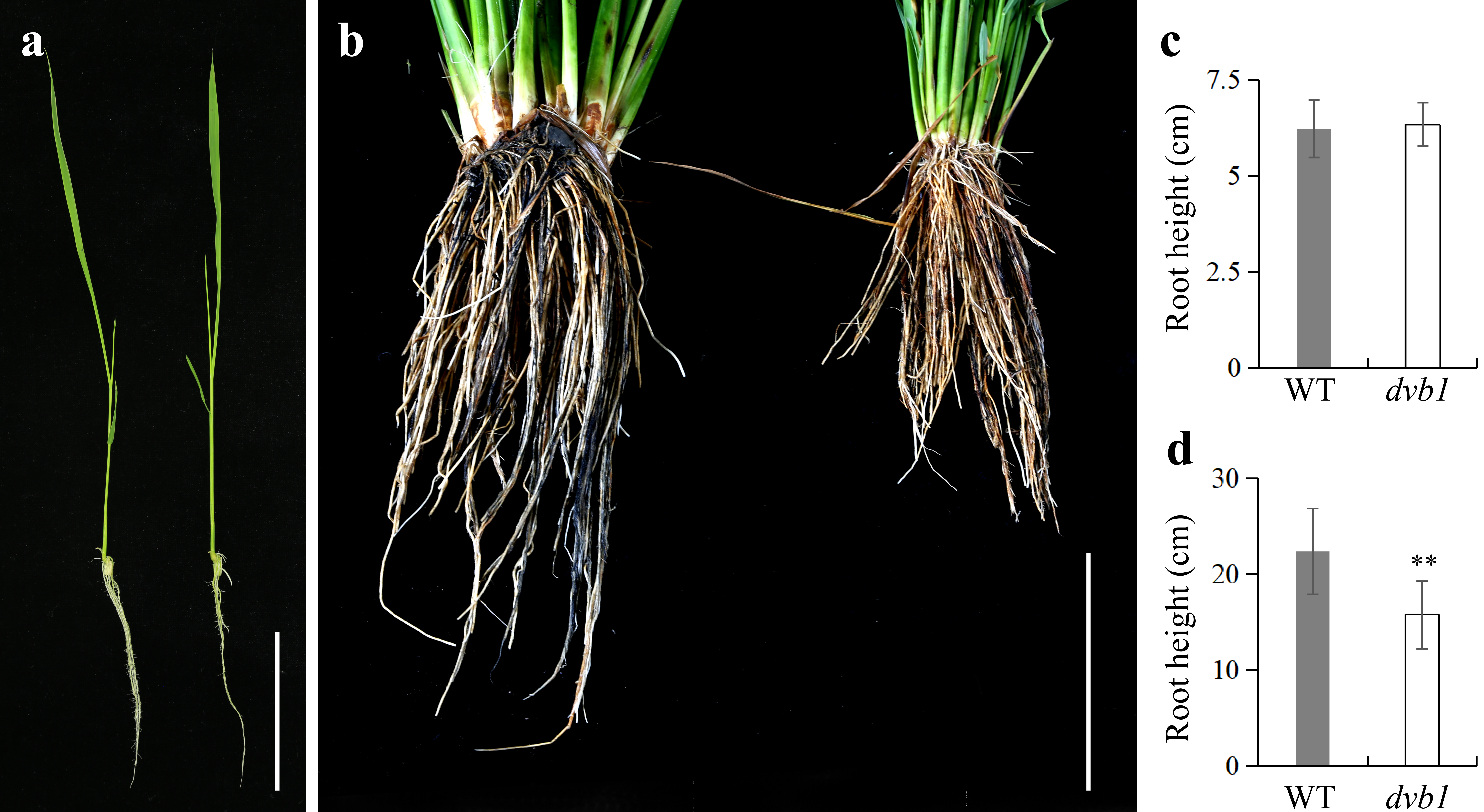


**Figure S1.** Root in *dvb1*

(**a**) The root in the wild type (left) and the *dvb1* mutant (right) at seedling stage. (**b**) The root in the wild type (left) and the *dvb1* mutant (right) at boosting stage. (**c**) Root length in the wild type and the dvb1 mutant at seedling stage. (**d**) Root length in the wild type and the *dvb1* mutant at boosting stage. Values represent means ± SD from 10 biological samples. Asterisks indicate the significance of differences between the wild type and *dvb1* as determined by Student’s *t*-test (*, 0.01 ≤ *P* < 0.05; **, *P* < 0.01). Scale bar: 5cm (**a**), 10cm (**b**).


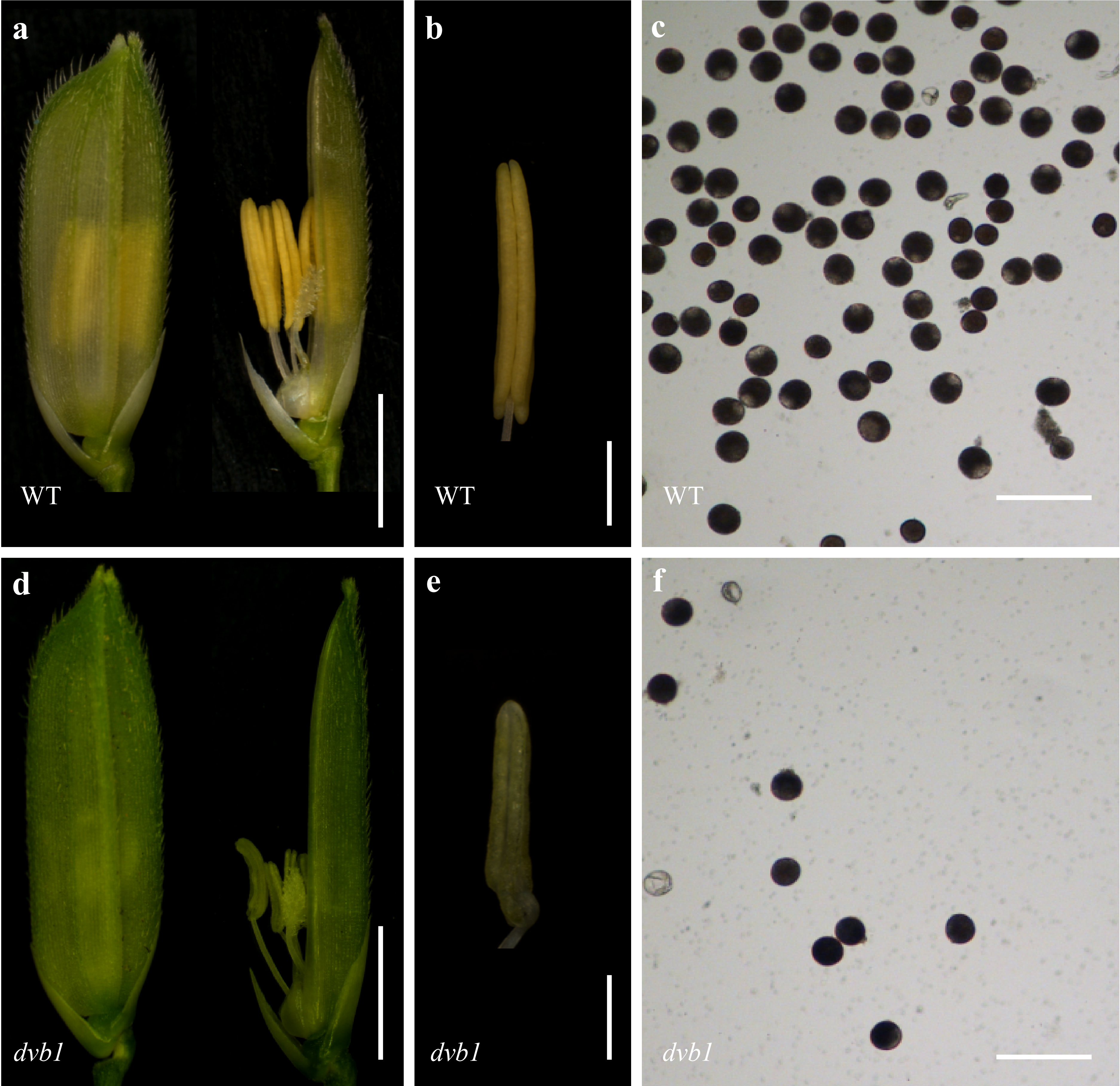


**Figure S2.** Anthers and pollens in *dvb1*

(**a**-**b**, **d**-**e**) The spike and anther of the wild type (**a**, **b**) and the *dvb1* mutant (**d**, **e**). (**c**, **f**) The KI-I_2_ staining of pollen in the wild type (**c**) and the *dvb1* mutant (**f**). Scale bar: 3 mm (**a**, **d**), 1mm (**b**, **e**), 200 μm (**c**, **f**).


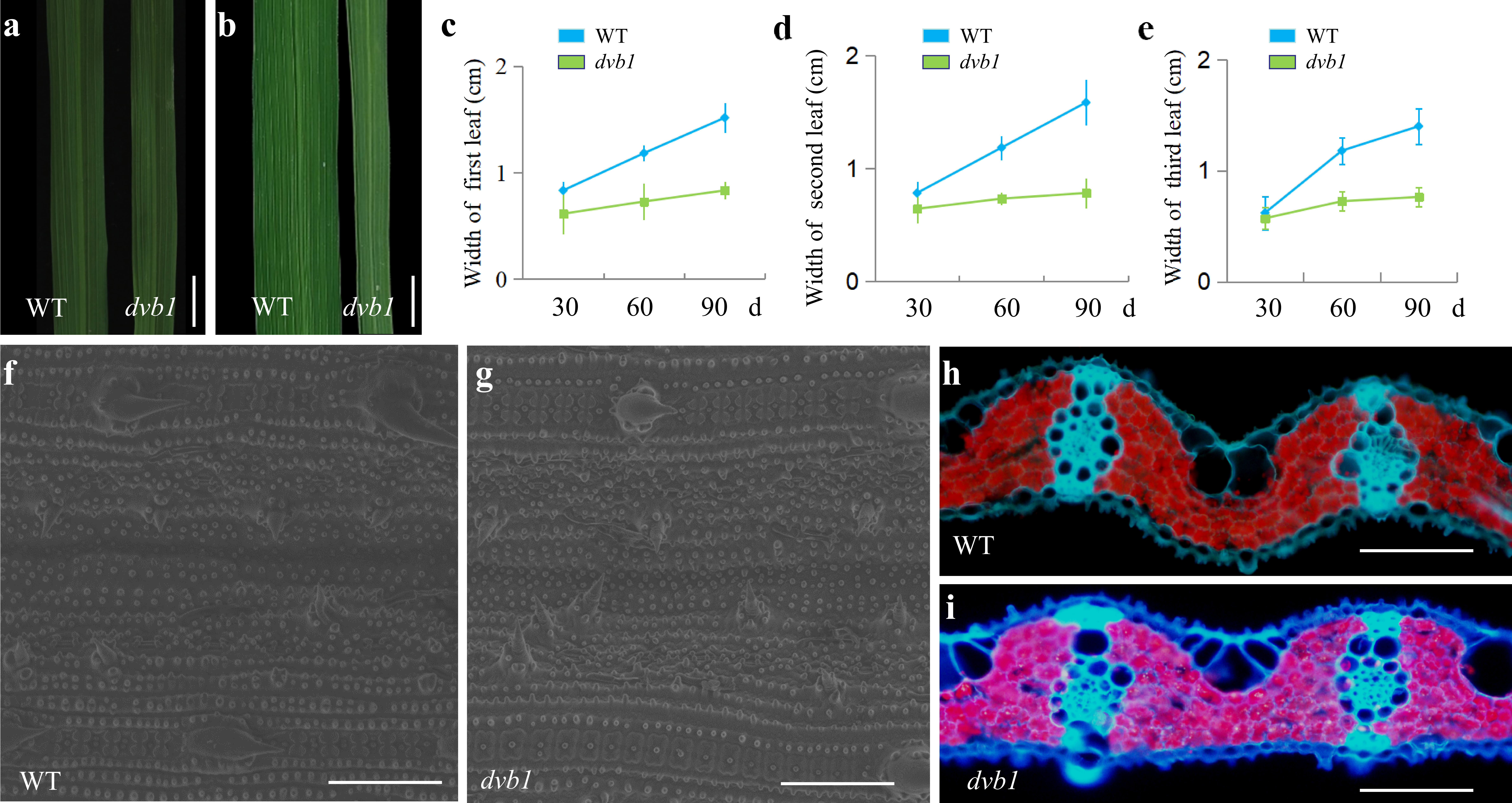


**Figure S3.** Narrowed leaves in *dvb1*

(**a**-**b**) The intermediate segment of the first leaf in the wild type (left) and the *dvb1* mutant (right) at earlier stage (**a**) and later (**b**) stage. (**c**-**e**) Widths of the intermediate segment of the first (**c**), second (**d**), third (**e**) leaf in the wild type and the *dvb1* mutant at day 30, day 60 and day 90 respectively. Values represent means ± SD from 10 biological samples. (**f**, **g**) Scanning electron microscopy of the leaves from wild type (**f**) and the *dvb1* mutant (**g**) plants. (**h**-**i**) Cryosectioning of the same part of leaves from wild type (**h**) and the *dvb1* mutant (**i**) plants. Scale bar: 1cm (**a**, **b**), 1μm (**f**, **g**), 200μm (**h**, **i**).


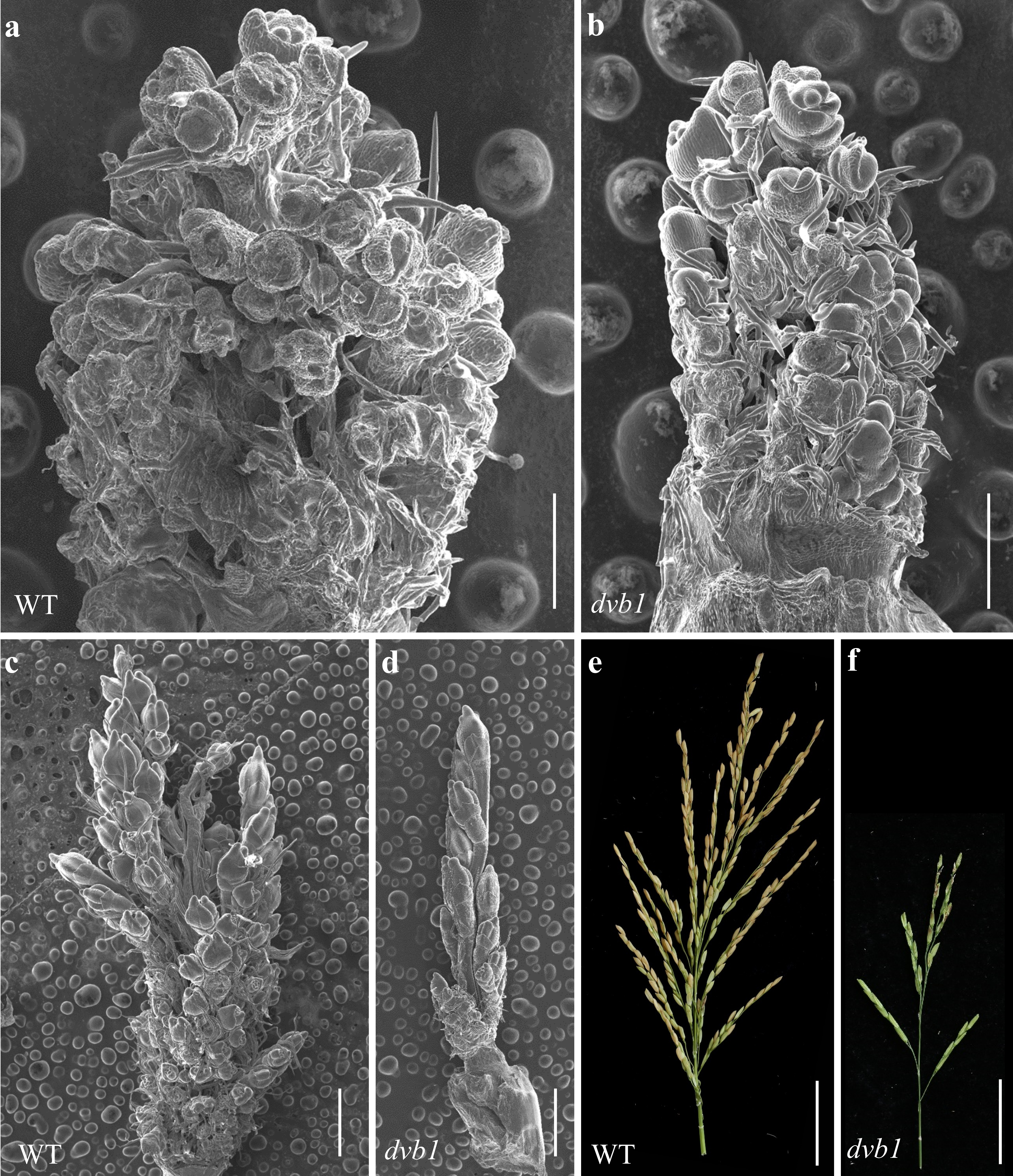


**Figure S4.** Flower primordia and branches in *dvb1*

(**a**-**d**) SEM of flower primordia and spikelets from wild type (**a**, **c**) and *dvb1* (**b**, **d**) plants. (**e**-**f**) Branches at mature spikes from wild type (**e**) and *dvb1* (**f**) plants. Scale bar: 40 μm (**a**, **b**), 20 μm (**c**, **d**), 3 cm (**e**, **f**).


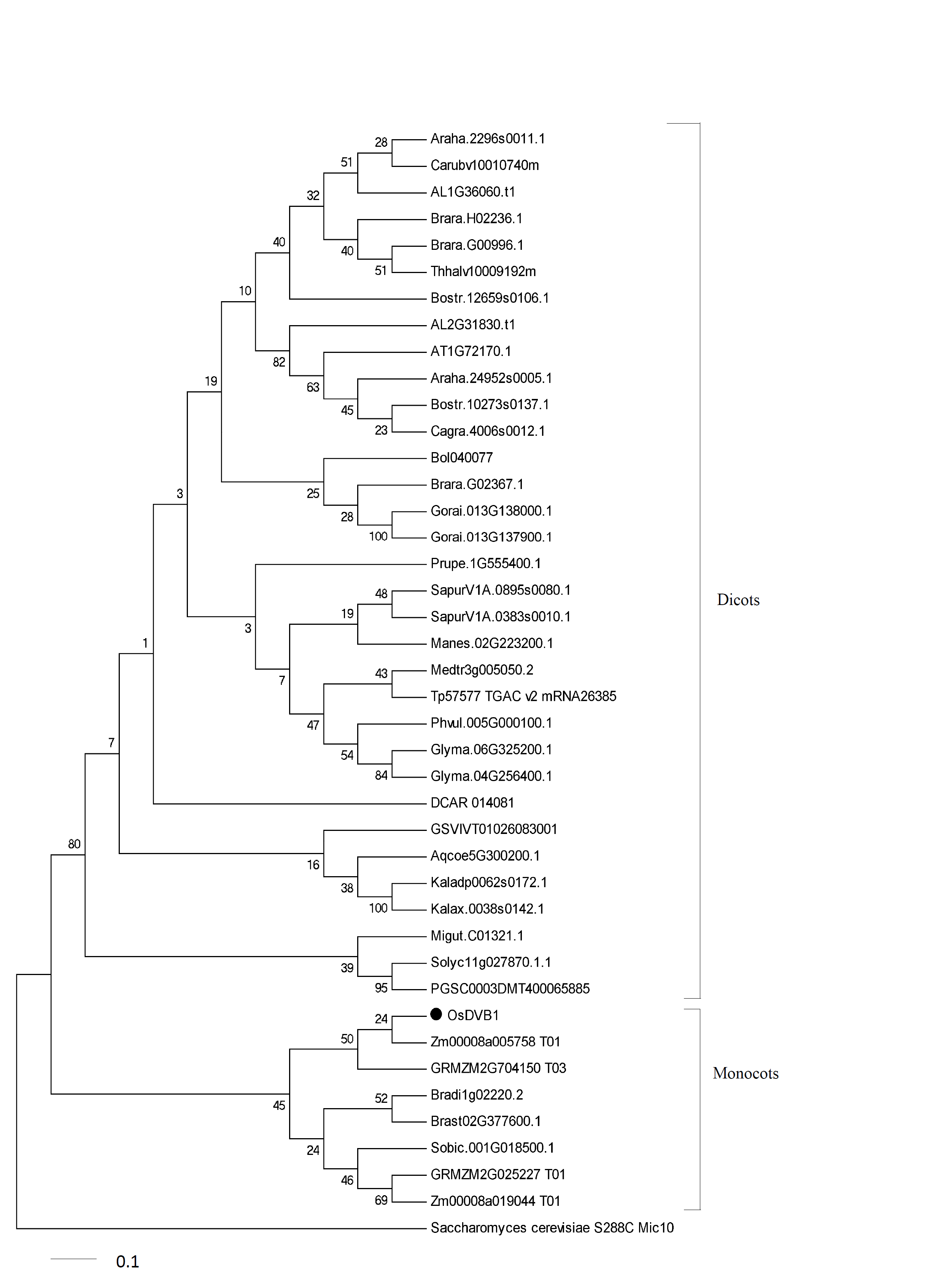


**Figure S5.** Phylogenetic tree of DVB1

Phylogenetic tree of DVB1 constructed by the neighbor-joining method. The number for each interior branch shows the percentage of the bootstrap value (1000 replicates). The scale bar indicates the estimated number of amino acid substitutions per site.


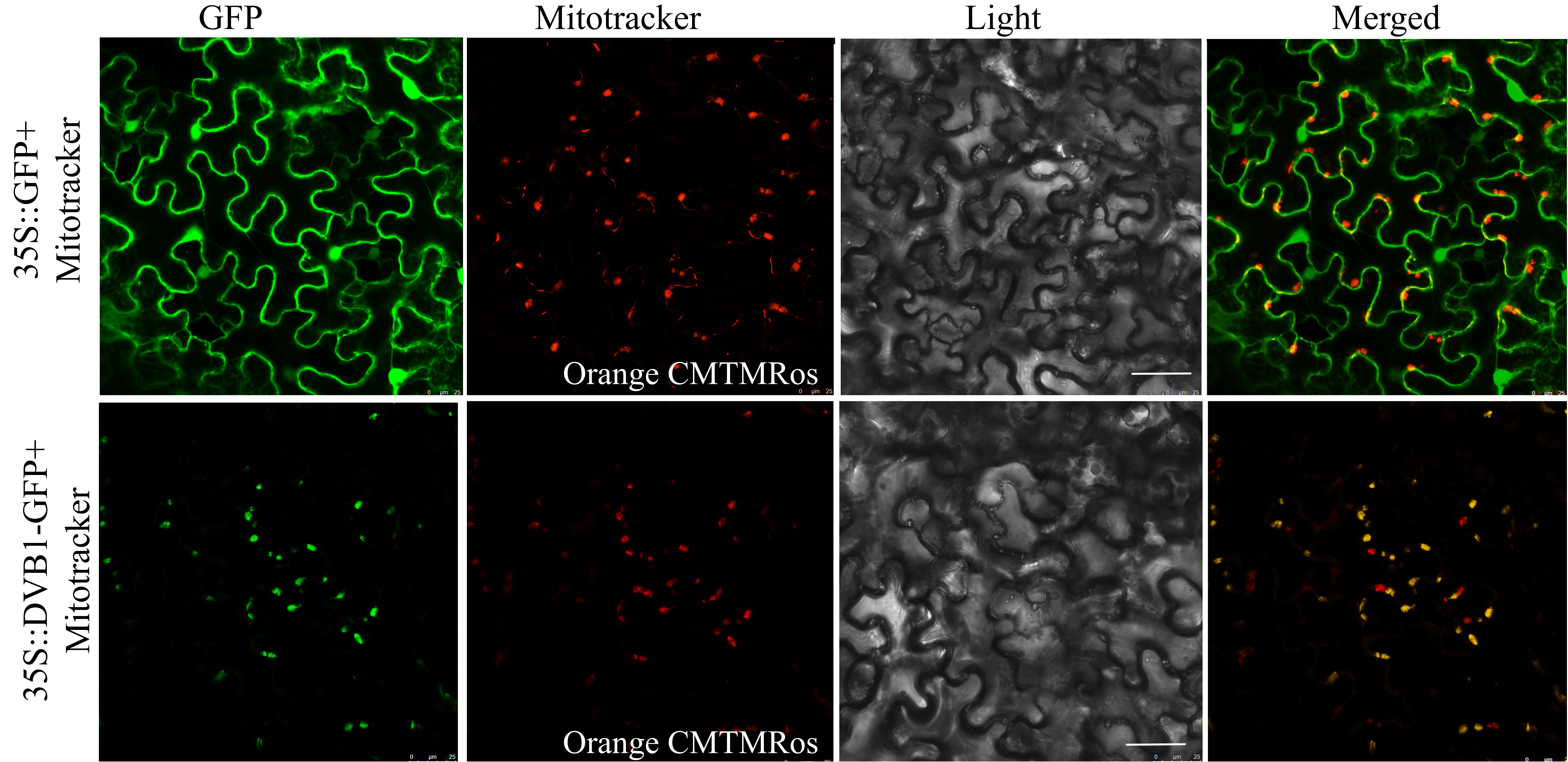


**Figure S6.** Subcellular localization of DVB1 protein in *Nicotiana benthamiana*

Subcellular localization of 35::GFP and 35::DVB1-GFP with MitoTracker Orange CMTMRos in *Nicotiana benthamiana.* Scale bar: 5 μm.


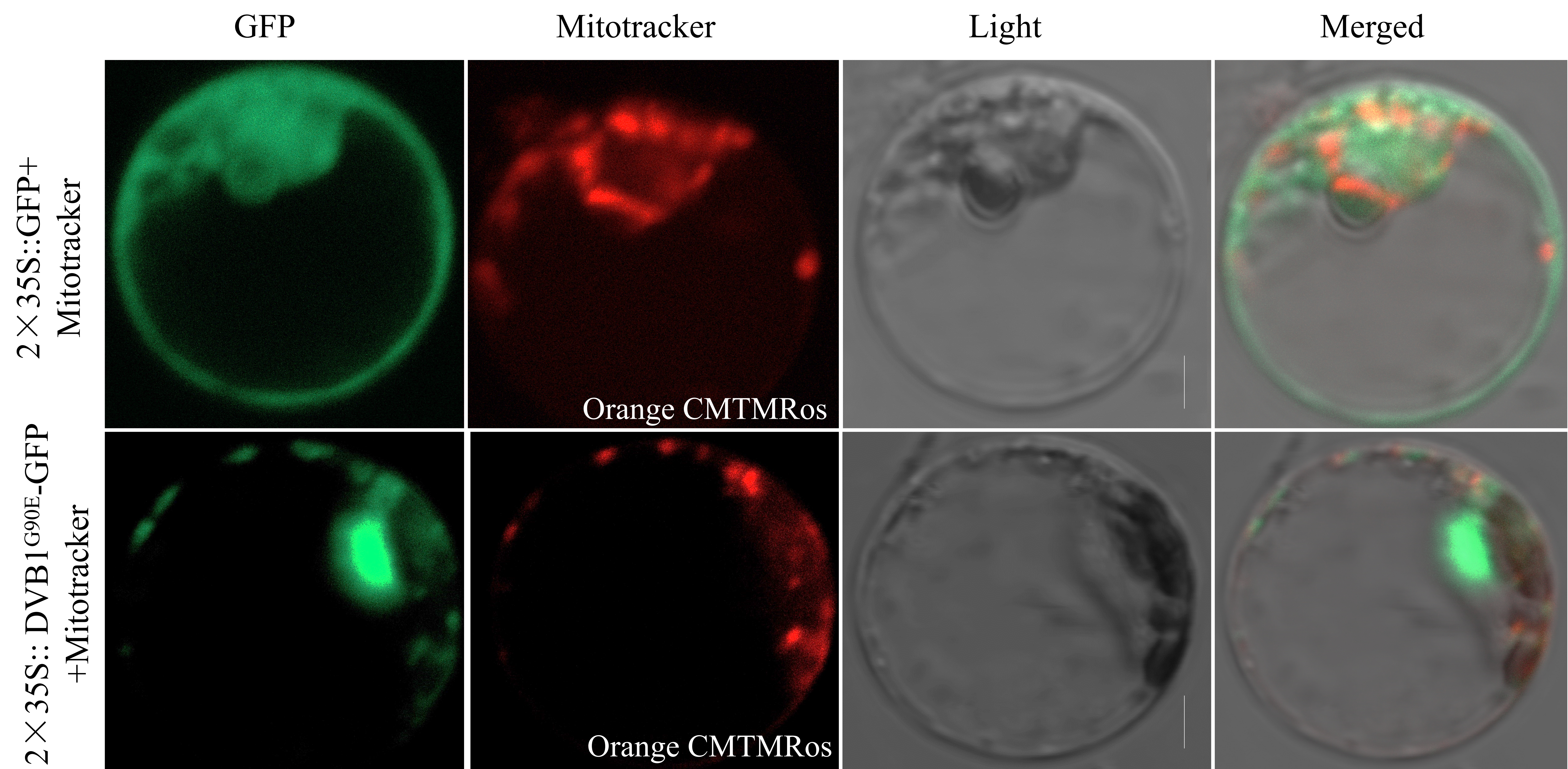


**Figure S7.** Subcellular localization of DVB1^G90E^ protein in rice protoplasts

Subcellular localization of 2×35S::GFP and 2×35S::DVB1^G90E^-GFP with MitoTracker Orange CMTMRos in rice protoplasts. Scale bar: 5 μm.

**Table S1.** Peptide sequences used for constructing the NJ tree

| ID | Peptides Sequences (N - C) |
| --- | --- |
| *OsDVB1* | MAESPENAAPAAAPAPAPAPTPAPAPPPPPSSPPTKSGIPPRYDLDAKWDACLDISIRRVAYSTLGGTFAGLLLFRSPTTRWASVALGAGVGIGAAYTECSYLFNGAPPKWSPKVSTVPSAHSEGEDK |
| *Bradi1g02220.2* | MAESPENAAAAPAPAPVPPPPAPKPSTPPPPPKSGIPPRYDLDAKWDACLDLSIRRVAYASLAGVFGGLILFRSPTTRWASVALGAGVGIGAAYTECSYIFNGSPPKWSPKVPSVPSAHSEGGDK |
| *Brast02G377600.1* | MAESPENAAAAPAPAPVPPPPAPKPSAPPPPPPKSGIPPRYDLDAKWDACLDLSIRRVAYASLAGAFGGLILFRSPTTRWASVALGAGVGIGAAYTECSYIFNGSPPKWSPKVPTVPSAHSEGDK |
| *Sobic.001G018500.1* | MAEAPETAAPTPPPSPPAPVTSSPPPKSGIPPRYDLDAKWDACLDLSIRRVAYSSLAGAFAGLLLFRSPTTRWASVALGAGVGIGAAYTECSYLFNGAPPKWSPKVSTIPSAHSEGGDK |
| *GRMZM2G025227_T01* | MAEATDTAAATPPPAPAMATLSPPPKSGIPPRYNLDAKWDACLDLSIRRVAYSSLAGALAGLLLFRSPTTRWASVTLGAGVGIGAAYTECSYLFSGGPPNWSPKVSTIPSAHSEVIALSFSLNPV |
| *GRMZM2G704150_T03* | MAEAPETAAPTQPPPAPAASSPPPKSGIPPRYDLDAKWDACLDLSIRRVAYSSLGGAFAGLLLFRSPTTRWASVALGAGVGIGAAYTECSYLFNGAPKCSPKVSTITSAHSDVIALSFSLNPV |
| *Zm00008a019044_T01* | MAEATDTAAATPPPAPAMATLSPPPKSGIPPRYNLDAKWDACLDLSIRRVAYSSLAGALAGLLLFRSPTTRWASVTLGAGVGIGAAYTECSYLFSGGPPNWSPKVSTIPSAHSEGEDK |
| *Zm00008a005758_T01* | MAEAPETAAPTQPPPAPAASSPPPKSGIPPRYDLDAKWDACLDLSIRRVAYSSLGGAFAGLLLFRSPTTRWASVALGAGVGIGAAYTECSYLFNGAPKCSPKVSTIPSAHSDVIALSFSLNPV |
| *Aqcoe5G300200.1* | MAEEKKQNPNRAFHVEYDLDAKWDECLDLTVRRSCYSSLAGAVTGLLFFRSPVTRWASVAFGAGIGIGSAYTECSQKFGGSFTKWTPPSKVPSTPASQGED |
| *DCAR_014081* | MAENKQIPAQYDVNAKWDACLDLGIRRFVYSSVGGAFAGLLLFRTPVTRWASVAFGAGLGIGSAYSECSTKFDGTSKKVEGLTSI |
| *Migut.C01321.1* | MVEVNKAEIPAKYDLNAKWDACLDLGLRRFVYASLSGGFAGLLLFRSPVTRWASVAFGAGVGIGSAYTDCSRKFGGSPTKLTDISDSSHSKAAEE |
| *Solyc11g027870.1.1* | MAEENKQEQSVVPSGYNLDAKWDACLDLGVRRFTYFSLIGGFAGLLLFRSPVTRWASTAFGAGAGLGSAYTECSQKFGGYPGKSTASISETPITKVGED |
| *PGSC0003DMT400065885* | MAEENKQEQSVVPSEYNLDAKWDACLDLGLRRFTYFSLIGGFAGLLLFRSPVTRWASTAFGAGAGLGSAYTECSQKFGGYPGKSTASISETPITKVGED |
| *Kaladp0062s0172.1* | MDPQQQRRPPIPQYDECLDLSLRRFVYSSLAGAFGGLLLFRSPVTRWASVAFGAGIGLGSAYTECNQKFNTSSVTLKATEEKVTENLASQDAQD |
| *Kalax.0038s0142.1* | MDPQQQRRPSIPQYDECLDLSLRRFVYSSLAGAFGGLLLFRSPVTRWASVAFGAGIGLGSAYTECNQKFNTSPVTLKATEEKVTENLASQDAQD |
| *GSVIVT01026083001* | MAENKQIPPPYDLDAKWDACLDLTVRRFVYSSFAGAFGGLLLFRSPVTRWASVAFGAGLGLGSAYTECSQKFGGYPAMFLPPK |
| *Manes.02G223200.1* | MAENHDINAKWDACLDLTVRRFVYSSLAGAFGGLLFFRTPATRWASAAFGAGVGIGSAYTECSRIFDGYPTKLASPKPSNAPAPES |
| *SapurV1A.0895s0080.1* | MAEKTDVNANWDACLDLSVRRFVYSSLAGAFGGLLLFRSPVSRWASVAFGAGVGIGSAYTDCSHIFGSPAKMECPKKTSSAHEQLTPLNTPHNVPASQDGQD |
| *SapurV1A.0383s0010.1* | MAEKSDVNAKWDACLDLSVRRFVYSSLAGAFAGLLLFRTPVTRRASIAFGAGVGIGSAYADCSRIFDGSAAKLVPPKTTSSAPEELQPPNSLSNPSQDGPGLRTDI |
| *Gorai.013G138000.1* | MGDDKELTPSKYDMNAKWDACIDLTLRRFVYSSLAGAFGGLLFFIVSMLFCTRQRWLFPLYMRLLYIHSCRCQCKYDVYTCPKYVGI |
| *Gorai.013G137900.1* | MGENKELTPSKYDMNAKWDACIDLTLRRFVYSSLAGAFVVSMLFCTRQRWLLQLYMRLLYIHSCRC |
| *Araha.24952s0005.1* | MAEKKDSLPPEYDVNAKWDACLDLTVRRFVYSSLGGAFAGLLFFRSPVTRWASIALGAGIGIGSAYTDCSRAFDAPSSSSANLTAPKNTTETSSVSQDADE |
| *Araha.2296s0011.1* | METTKSNNTDSDVNAKWDACLDLTARRFIYSSLGGAFAGLLFFRSPVTRWASIAFGAGIGIGSAYTDCSRVFDASSSTSATLLAAPKSTETSVSQAAEE |
| *AL1G36060.t1* | METTKSNNTDSDVNAKWDACLDLTARRFVYSSLGGAFSGLLFFRSPVTRWASIAFGAGIGIGSAYTDCSRVFDASSSTSATLLAASKSTETSVSQAAEE |
| *AL2G31830.t1* | MAEKNGSLPPEYDVNAKWDACLDLTVRRFVYSSLGGAFAGLLFFRSPVTRWASIALGAGIGIGSAYTDCSRSFDAPSSSSANLAAPKDTTETSPVSQAAEE |
| *AT1G72170.1* | MAEKKDSVPPEYDVNAKWDACLDLTVRRFVYSSLGGAFAGLLFFRSPVTRWASIALGAGIGIGSAYSDCSRAFDSPSSSSANLAAPKNNITETSPVSQAADE |
| *Bostr.12659s0106.1* | METKSNNTESDVNAKWDACLDLTTRRFVYSSLGGAFAGLLFFRSPVTRWASIAFGAGIGIGSAYTDCSRVFDGSSSTSATLLAPKSSTETSVSQAAEE |
| *Bostr.10273s0137.1* | MAEKKDIVPPDYDVNAKWDACLDLTVRRFVYSSLGGAFAGLLFFRSPVTRWASIALGAGIGIGSAYTDCSRAFDTPSSPSYSLAVPNNTTETSASQVSWNLFWQMH |
| *Bol040077* | MEERKENVPPEHDVNAKWDACIDHATRRFVYSSLGGAFAGLLFFRSPVTRWASIAFGAGLGIGSAYTDCSRAFDAPSSSSSSSPAAPKSRETSSVSQVSWHHFLTNAFVW |
| *Brara.G00996.1* | MEKKSNGESDVNSKWDACIDITARRVVCSSLGAAFAGLLFFRSPVTRWASTACCAGIGMGSAYADCSRFFDSSSSATSPTSTETSYFVFQAAEEQEKEEA |
| *Brara.G02367.1* | MGEGKESVPQEHDVNAKWDACIDLTTRRFVYSSLGGAFAGLLFFRSPVTRWASIAFGAGLGIGSAYTDCSRAFDAPPSFTETSSVSQTVSQSADE |
| *Brara.H02236.1* | MEKKKSDDVNSKWDACLDLTARRVVYSSLGGAFAGLLFFRSPVTRWASIAFGAGLGIGSAYTDCSRVFDASSSSSASAALVAPISTETSSSSSYVSQAEEE |
| *Cagra.4006s0012.1* | MAEKKGAVPPDYDVNAKWDACLDLTVRRFVYSSLGGAFAGLLFFRSPVTRWASIALGAGIGIGSAYTDCSRAFDAPSSSSPNLASPKSTPKTSVSQGADE |
| *Carubv10010740m* | METKSNNTDVNAKWDACLDLTARRFVYSSLGGAFAGLLFFRSPVTRWASIAFGAGIGIGSAYTDCSRVFDASSSTSATLLAPKTSTETPVSQAAEE |
| *Thhalv10009192m* | MEKKSNSESDVNAKWDTCLDLTARRVVYSSLGGAFAGLLFFRSPVTRWASIAFGAGIGIGSAYADCSRVFNTSSSTSATLLAPKSTETSVSQAAEE |
| *Glyma.06G325200.1* | MAEKKEMIPQQFDIDAKWDACIDLTVRRFVYSATAGAFGGLLFFRSPVTRWASIAFGAGVGIGSAYAECSRLFDGPPTNLPLPKVSETATQDSD |
| *Glyma.04G256400.1* | MAEKKEMIPKQYDIDAKWDACIDLTVRRFVYSASAGAFGGLLFFRSPVTRWASIAFGAGVGIGSAYAECSRLFDGPPTNLPLPKVSETATQNVPDSE |
| *Medtr3g005050.2* | MGENKEELIPQKYDVDAKWDACLDLTVRRFVYSSFAGAFGGLLLFRSPQTRWASIAFGAGVGIGSAYAECSRLFDGSPAKLAQHKDSEAPTQLFFLVLL |
| *Phvul.005G000100.1* | MADKQIIPPPHDLDAKWDACLDLTVRRFVYSSTAGAFAGLLFFRSPVTRWASIAFGAGVGIGSAYAECSRLFDGPPTKLPLPKVSETAAQNVQVSE |
| *Prupe.1G555400.1* | MADNKEFFPPQYDVNAKWDACLDLTVRRFVYSSLGGAFGGLLLFRSPVSRWASVAFGAGLGIGSAYTECSRLFEGSPAKLAAPKIIETPAPQHNSSSDNLVI |
| *Tp57577_TGAC_v2_mRNA26385* | MADNKEIIPRHYDLDAKWDACLDLTVRRFVYSSFAGAFGGLLLFRSPQTRWASIAFGAGVGIGSAYTECSRLFDGPPTKLALPKGSDAPIQNTQDSE |
| *Saccharomyces cerevisiae S288C Mic10* | MSEQAQTQQPAKSTPSKDSNKNGSSVSTILDTKWDIVLSNMLVKTAMGFGVGVFTSVLFFKRRAFPVWLGIGFGVGRGYAEGAIFRSSAGLRSSKV |

**Table S2.** Primer sequences used in this study

| Primer Name | Sequence (5' - 3') | Purpose |
| --- | --- | --- |
| *DVB1* Com-F | ACGAATTCGAGCTCGGTACCGTATGCATTTGTGTGTGTTGACCCAGCA | Complementary of *dvb1* |
| *DVB1* Com-R | CGACGGCCAGTGCCAAGCTTGAAACACAAGGAGTGGCCTAAACG |
| *DVB1* RT-F | GTACACTGAATGTTCATACTTATTCAATG | qRT-PCR |
| *DVB1* RT-R | CCTTCAGAATGAGCAGAAGGAA |
| *DVB1* ISH-F | ACCAAGTCGGGGATCCC | *In situ* analysis of *DVB1* |
| *DVB1* ISH-R | TAATACGACTCACTATAGGGTATGAACATTCAGTGTACGCAG |
| *DVB1*/ *DVB1^G90E^*580-F | GACAGCCCAGATCAACTAGTATGGCGGAGTCGCCCGAG | Subcellular localization of DVB1 |
| *DVB1/ DVB1^G90E^* 580-R | CCCTTGCTCACCATGGATCCCTTGTCTTCCCCTTCAGAATGAGCA |
| *DVB1* 1300-F | GAACACGGGGGACGAGCTCGGTACCATGCAGGAGTCGCCCGACGAC |
| *DVB1* 1300-R | CCTTGCTCACCATGTCGACTCTAGACTTGTCTTCCCCTTCAGAATG |
| *DVB1/DVB1^G90E^* AD/BD  -F | GGAATTCCATATGATGGCGGAGTCGCCCGA | Yeast two-hybrid |
| *DVB1/DVB1^G90E^* AD/BD  -R | CCGGAATTCTTACTTGTCTTCCCCTTCAGAATGAGC |
| *DVB1^1-91AA^* AD/BD  -F | GGAATTCCATATGATGGCGGAGTCGCCCGA |  |
| *DVB1^1-91AA^* AD/BD  -R | CCGGAATTCGCGAGTAGTTGGGCTACGGAA |  |
| *DVB1/DVB1^G90E^* 32a-F | ACAGCCCAGATCTGGGTACCATGGCGGAGTCGCCCGA | Protein expression |
| *DVB1/DVB1^G90E^* 32a-R | TGTCGACGGAGCTCGAATTCTTACTTGTCTTCCCCTTCAGAATGAGC |
| *OsFIB* RT-F | CTTCAAAGGCGACTCCTACATTGAG | qRT-PCR |
| *OsFIB* RT-R | GTGCTCTTGGAGACGGTGAAGAG |
| *OsTAR1* RT-F | GAGGTGCGCCGCCTCCA |
| *OsTAR1* RT-R | GAGCGCGGCCTGGAAGAG |
| *OsTDD1* RT-F | GAGGTATATCGCAATGATGAACTTACCA |
| *OsTDD1* RT-R | TCCAGAACAGTCTGCAATGATATACCT |
| *OsCOW1* RT-F | CATGTTCTACCGAGAGAGATGTTTGG |
| *OsCOW1* RT-R | GCCTCTTGAGACCATACTTCTCGG |
| *ACTIN1* RT-F | GACCCAGATCATGTTTGAGACCT |
| *ACTIN1* RT-R | CAGTGTGGCTGACACCATCAC |
